# Soil contamination by metals and radionuclides in missile-affected areas: radiological risk assessment and phytotoxic effects on wheat plants

**DOI:** 10.1007/s10653-026-03375-6

**Published:** 2026-07-27

**Authors:** Ewa Skwarek, Lydia Babenko, Oksana Futorna, Lesya Voytenko, Małgorzata Wiśniewska, Valentyna Vasyuk, Sergiy Melnychuk, Volodymyr Korotaiev, Victoria Paientko, Iryna Kosakivska, Iwona Ostolska

**Affiliations:** 1 Department of Radiochemistry and Environmental Chemistry, Institute of Chemical Sciences, Faculty of Chemistry, Maria Curie-Sklodowska University in Lublin, M. Curie-Sklodowska Sq. 3, 20-031 Lublin, Poland; 2 M.G. Kholodny Institute of Botany, National Academy of Sciences of Ukraine, 2 Tereshchenkivska St., Kiev, 01004 Ukraine; 3 Educational and Scientific Institute of High Technologies, Taras Shevchenko National University of Kyiv, 4-G Hlushkova Avenue, Kiev, 03022 Ukraine; 4 Private Joint-Stock Company “Myronivsky Hliboproduct”158, AkademikaZabolotnoho St, Kiev, 03143 Ukraine; 5 D. K. Zabolotny Institute of Microbiology and Virology of the National Academy of Sciences of Ukraine, 154, AkademikaZabolotnoh St, Kiev, 03143 Ukraine; 6 Dnipropetrovsk Scientific Research Forensic Centre of the Ministry of Internal Affairs of Ukraine, 1 BudivelnyiTupyk, Dnipro, 49000 Ukraine; 7 Institute of Surface Chemistry, National Academy of Sciences of Ukraine, 17 General Naumov Street, Kiev, 03164 Ukraine

**Keywords:** Soil, Gamma-ray spectrometry, Radiological hazard indices, Metals pollution, Morphophysiological responses, *Triticum aestivum*

## Abstract

Military conflicts constitute an increasingly important yet still insufficiently quantified source of environmental contamination in agricultural ecosystems. This study investigated soils affected by missile strikes during the Russian aggression in Ukraine, with particular emphasis on heavy metal accumulation, radionuclide occurrence, and associated phytotoxic effects on wheat (*Triticum aestivum L*.). Geochemical analyses using X-ray fluorescence spectroscopy revealed pronounced enrichment of Pb, Zn, Cu, Cr, Ni, and Mn in crater soils, frequently exceeding local geochemical background levels and environmental guideline thresholds. Elevated activity concentrations of ^137Cs were additionally detected, indicating contamination associated with explosive materials and projectile components. Despite locally increased radionuclide levels, the calculated radiological indices demonstrated that the investigated soils do not currently pose a significant radiological hazard to human health. The contamination was accompanied by soil acidification, compaction, and degradation of physical structure, suggesting long-term disturbance of soil functioning in affected agricultural areas. Among the detected contaminants, Pb was consistently enriched in crater soils and was therefore selected as a representative model toxicant to investigate the biological mechanisms linking field-observed contamination to crop responses under controlled conditions. Hydroponic experiments demonstrated that Pb exposure induced a clear, dose-dependent inhibition of plant growth, biomass accumulation, and stress tolerance indices, whereas low Pb concentrations produced a slight hormetic response. Anatomical analyses of wheat roots revealed substantial structural reorganisation, including reduced stele and xylem development, enhanced cortical porosity, increased endodermal suberisation and lignification, and reduced vessel diameter, potentially limiting water and nutrient transport. Strong correlations between Pb concentration, anatomical modifications, and morphophysiological responses indicate that root structural disruption represents a major mechanism of toxicity. The results demonstrate that missile-derived contamination may significantly affect soil quality and crop performance even outside active combat zones. Furthermore, the study highlights the usefulness of root anatomical traits as sensitive biomarkers for early detection of military-induced soil stress and provides new insight into the combined, radiological and mechanistic biological approaches for assessing post-conflict agricultural environments.

## Introduction

Metals pollution is one of the most persistent and dangerous forms of human-made environmental impact, posing serious threats to both ecosystems and human health (Kosakivska et al., 2021; Yao et al., 2024; Liu et al., 2026). Unlike organic contaminants, metals are not biodegradable; they tend to accumulate in soils, bottom sediments, and living organisms and can remain in the biosphere for decades. That chronic exposure, even at low concentrations, is linked to neurotoxic effects, cancer risk, reproductive issues in humans, as well as biodiversity loss and reduced soil fertility (Priyadarshanee et al., 2022; Wan et al., 2024).

Military activity is a significant but often underestimated source of metals put into the environment. Explosive materials, armour-piercing ammunition, combustion products from military vehicle engines, and the destruction of industrial facilities release potentially toxic elements such as Pb, Cu, Zn, Ni, Cr, and Mn, and, in some cases, even depleted uranium (Skalny et al., 2021). Unlike pollution from conventional industrial or agricultural sources, contamination resulting from military actions exhibits a pronounced spatial gradient, is highly intense over time, and often overlaps with pre-existing anthropogenic pollution, thereby substantially complicating environmental monitoring and risk assessment (Broomandiet al., 2020).

Among natural sources of ionizing radiation, radon (^222^Rn) and its short-lived decay products present the most significant contribution to the inhalation dose received by the human population (WHO, 2009). ^222^Rn is a naturally occurring alpha-emitting radioactive gas formed within the uranium (^238^U) decay series, directly originating from the alpha decay of ^226^Ra present in soil and rocks. Due to its inert chemical nature and physical properties, radon can easily migrate through the soil pore space via diffusion and advection, subsequently exhalating into the atmosphere or accumulating in enclosed indoor environments (Kouroukla et al., 2024). Inhalation of radon progeny, specifically ^218^Po and ^214^Po, which tend to deposit in the human respiratory tract, poses a severe health hazard and represents the second leading cause of lung cancer globally after tobacco smoking (Darby et al., 2005).

Russia’s full-scale invasion of Ukraine, launched in 2022 against the backdrop of an ongoing conflict since 2014, has caused serious environmental damage. As of October 2024, about 18% of Ukraine’s territory remains under occupation. Environmental damages are estimated to exceed USD 56.4 billion. Around 30% of the country’s land is polluted with landmines and unexploded ordnance, while negative impacts such as landscape destruction, wildfires, deforestation, and pollution have affected 30% of Ukraine’s protected areas (Baliuket al., 2024; Hlavatskyi et al., 2025; Leal Filho et al., 2024a,b). The occupation of the Zaporizhzhia Nuclear Power Plant and the destruction of the Kakhovka Dam have increased the risk of long-term environmental catastrophe (Hryhorczuk et al., 2024).

Large-scale artillery shelling, missile strikes, and infrastructure destruction are accompanied by the direct release of metals into soil, air, and aquatic ecosystems (Leal Filho et al., 2024a, b; Bilyiet al., 2025). Preliminary studies have reported elevated concentrations of Cr, Zn, Pb, and other elements in explosion craters, surface waters, and agricultural soils. In particular, in the Luhansk region, background concentrations were exceeded by 7.6 times for Zn, 1.4 times for Cd, and 1.1 times for Pb (Yakymchuk et al. 2024). Soil analysis conducted in Lviv following a missile strike revealed exceedances of the maximum permissible concentrations (MPCs) for Ti, Zn, Pb, and Ni in all samples; notably, plants demonstrated efficient uptake of Zn, Cu, Cr, and Cd (Petrushka et al., 2024). In the Kharkiv region, Zn concentrations exceeded background levels threefold and MPC values by 1.2–1.4 times, while the mean Cu concentration reached 1.45 MPC (Krainiuk et al., 2025). According to estimates by the Ukrainian Nature Conservation Group, shelling of just one square kilometre of agricultural land in the Kharkiv region can introduce approximately 50 tonnes of iron, 1 tonne of sulphur compounds, and 2.35 tonnes of copper into the soil (Munitions and Chemicals, 2022 https://rubryka.com/en/article/soil-ukraine/).

Landscape destruction, mechanical soil compaction caused by heavy military equipment, contamination with missile, drone, and ammunition fragments, as well as fuel residues, lead to changes in soil structure, disruption of the microbiome, and increased toxicity. The accumulation of metals in the upper soil layers contributes to physicochemical degradation and negatively impacts agroecosystems (Solokha et al. 2024; Illienkoet al., 2025). Concentrations of Pb, Hg, Cd, As, Cu, Cr, and Zn significantly exceed safe threshold levels in active combat zones, posing serious risks to food security and the health of millions of people (Broomandi et al., 2020).

Pb is one of the dominant and most stably accumulated HMs in soils following anthropogenic and military impacts. It is classified as a priority pollutant in international regulatory frameworks, in particular under European Union water policies (Directive 2000/60/EC; Directive 2013/39/EU) and in the United States Environmental Protection Agency's list of toxic substances (Priority Pollutants List C). The high level of environmental hazard posed by Pb is further confirmed by assessments from the World Health Organisation and the Food and Agriculture Organisation, which categorise it as a globally significant toxicant with pronounced bioaccumulation and long-term persistence in soil and aquatic ecosystems. Owing to these properties, lead is considered a key indicator of anthropogenic pressure and environmental degradation. Under natural conditions, Pb interacts with other metals, potentially altering its toxicity (Gupta et al., 2024; Rani et al., 2024a,b). Using Pb as a model element facilitates the identification of fundamental mechanisms behind its phytotoxic effects. These mechanisms are well documented; specifically, Pb exposure has been shown to inhibit cell division, weaken cell wall integrity, and obstruct apoplastic transport through increased suberisation and lignification of the endodermis. This enables reliable comparisons between observed results and published data (Kaur et al., 2013; Fahr et al., 2013; Kosakivska et al., 2025).

Despite the gradual accumulation of data, the effects of missile strike outside active combat zones, that is, areas where agricultural activities continue, remain poorly understood. The ecological significance of our study lies in its focus on agro-landscapes rather than zones of direct frontline impact, where land use is actively maintained or restored after missile strikes. Such areas pose a particular challenge for environmental monitoring, as visual signs of destruction may be partially erased. At the same time, chemical contamination and changes in soil physicochemical properties can persist for extended periods.

The rationale for our study involved a sequential process of several stages. First, local soil pollution in explosive-metal craters was assessed, with Pb identified as one of the most consistently elevated elements. Subsequently, Pb was used as a model toxicant to simulate the potential phytotoxic effects of post-bombing pollution under controlled conditions, and the morphophysiological and anatomical responses of wheat seedlings were examined. This approach enabled us to identify not only the growth inhibition but also its structural basis, specifically the reorganisation of essential tissues. We believe this method facilitates a shift from simply documenting contamination to understanding its possible biological mechanisms. In this context, using *T. aestivum* as a test system allows extrapolation of the findings to real agricultural systems. Now, bread wheat is one of the most strategically important cereal crops globally. As a staple commodity, wheat supports global food security. Ukraine has been among the leading exporters of wheat, accounting for approximately 9–10% of global wheat exports before the full-scale invasion. The disruption of Ukrainian agricultural production due to the war will therefore have systemic impacts on global food supply chains (FAO, 2022). According to FAO assessments, the conflict has increased global food insecurity, with projections suggesting that an additional 11–19 million people may face chronic hunger due to reduced grain exports (Welsh, 2024). Against this backdrop, studies using bread wheat as a model organism gain not only agronomic but also socio-economic importance. Any factor affecting wheat productivity, quality, or environmental safety, such as pollution from missile strikes, has implications that reach beyond local agroecosystems to global food security. Consequently, the current research adds to an increasing body of evidence connecting conflict-related environmental disruptions with threats to agricultural sustainability and, ultimately, to the stability of global food systems.

Thus, this study aims to assess the impact of missile strikes on soil physicochemical properties and the accumulation of metals in impact craters outside active combat zones. In addition, it seeks to elucidate the morphophysiological and anatomical mechanisms underlying the phytotoxic effects of Pb, used as the primary model toxicant, on *T. aestivum* seedlings. Notably, this study is the first in Ukraine to investigate the environmental consequences of ballistic missile strikes beyond active combat zones.

## Materials and methods

Soil analyses were conducted from 2024 to 2025 at the Department of Radiochemistry and Environmental Chemistry, Faculty of Chemistry, Institute of Chemical Sciences, Maria Curie-Sklodowska University (Lublin, Poland). Biological hydroponic experiments with wheat plants were carried out from 2024 to 2025 at the Department of Phytohormonology, M. G. Kholodny Institute of Botany, National Academy of Sciences of Ukraine (Kyiv, Ukraine).

### Study area and soil sampling

Soil samples were collected from agricultural lands in the Dnipropetrovsk region of Ukraine. All study sites were located outside active combat zones but had been affected by ballistic missile strikes. Before the strikes, the sites had no history of industrial activity or military use and were used exclusively for agricultural production. All soil samples were collected simultaneously in early 2024 to ensure comparability among the investigated sites. Due to ongoing security concerns related to military activity in the region, the exact geographic coordinates and a detailed map of the sampling locations are not provided. Instead, the study sites are described in a generalized manner sufficient to ensure reproducibility in terms of soil type and environmental conditions while preventing precise site identification. At each site, five soil subsamples (300 g each) were collected from the upper soil horizon (0–20 cm) and combined to produce a single homogenized composite sample representative of the site. Control soils were collected 550–600 m from the crater centre, in the direction opposite to the prevailing wind, to minimise the risk of direct contamination while ensuring comparable soil type, topography, and previous land use. Pre-strike soil measurements were unavailable; therefore, the control samples were used as local background references rather than as absolute pre-strike baselines. Because fine particulate matter generated during explosive events may have been redistributed beyond the immediate impact zone, these control samples do not represent a true geochemical baseline. Consequently, the calculated enrichment factors and geoaccumulation indices should be regarded as conservative estimates of contamination intensity. Prior to analysis, the composite soil samples were air-dried at room temperature and divided into subsamples. One subsample was used to determine pH and particle-size distribution, whereas another was gently ground and then sieved through a 1-mm mesh sieve to obtain a homogeneous, fine powder for elemental analysis by energy-dispersive X-ray fluorescence (ED-XRF, model Axios mAX, PANalytical, Holand) and gamma-ray spectrometry. Each composite sample was analysed in five analytical replicates, and the mean value of these replicates was used as the representative value for the corresponding sampling site. Characteristics of the sampling sites are presented in Table 1.

**Table 1:** Characteristics of the soil sampling sites, including site description, soil pH, and mean particle size

Site ID	Sampling site description	pH, mean ± SD	Mean particle size (µm), mean ± SD
1	Control site for Site 1′	8.54 ± 0.31	720.35 ± 1.02
1′	Explosion crater (25 May 2022)	8.91 ± 0.01	1214.81 ± 5.10
2	Control conditions correspond to Site 1	9.00 ± 0.02	986.11 ± 5.21
2′	Direct missile impact (30 Nov 2023; crater in an outbuilding on a private residential property near Site 1′)	8.85 ± 0.09	1140.54 ± 15.52
3	Control site for Site 3′	8.78 ± 0.13	947.12 ± 5.15
3′	Explosion crater (23 Nov 2023)	7.78 ± 0.03	1062.54 ± 7.21
4	Control site for Site 4′	9.32 ± 0.06	841.21 ± 5.33
4′	Explosion crater (29 Aug 2022)	7.20 ± 0.02	1113.64 ± 6.13
5	Control site for Site 5′	8.24 ± 0.01	1006.88 ± 6.05
5′	Explosion crater (15 Jul 2022)	8.78 ± 0.19	1129.73 ± 3.14
6	Control site for Site 6′	7.60 ± 0.02	946.39 ± 5.67
6′	Explosion crater (03 Sep 2023)	7.85 ± 0.03	1246.01 ± 6.04
7	Control site for Site 7′	7.61 ± 0.01	947.65 ± 6.23
7′	Explosion crater (24 Jun 2023)	7.20 ± 0.01	1069.93 ± 2.58

Sites marked with a prime symbol (′) represent missile impact locations (explosion craters), whereas sites without the prime denote the corresponding control plots. Site 1 served as the common control for both impact sites (Sites 1′ and 2′); therefore, a separate control site (Site 2) was not established. Values are presented as mean ± standard deviation (SD) of five analytical replicates obtained from a homogenized composite soil sample prepared by combining five field subsamples collected at each site

### Gamma – ray spectrometry and radiological hazard assessment

The concentration of radionuclides emitting gamma rays was determined using a gamma-ray spectrometer. It is evident that these nuclides, given their propensity to emit radiation and their pervasive occurrence, may pose a significant threat to living organisms. It is important to note that high activity levels of isotopes classified as NORM (Naturally Occurring Radioactive Materials) have the potential to contribute to the receipt of high doses of gamma radiation.

Prior to gamma spectrometry measurements, all collected soil samples were meticulously cleared of visible debris, including stones, root fragments, plant matter, glass, and plastic. The cleaned material was subsequently dried at a temperature below 100 °C and sieved through a 1-mm mesh to ensure homogeneity. Next, a portion of each homogenized sample was weighed using an analytical balance (accuracy of ± 0.01 g) and transferred into a 450-mL Marinelli beaker. To prevent the escape of gaseous radon-222, the beakers were hermetically sealed using a layer of metallized tape, further reinforced with construction adhesive tape. The sealed containers were then stored for a minimum of 4 weeks to allow the secular radioactive equilibrium between ^222^Rn and its short-lived decay products to be established. Following the equilibration period, the samples were placed in the measurement chamber of a gamma spectrometry system. Gamma-ray spectra were acquired using a high-purity germanium (HPGe) detector with coaxial geometry (model GEM40-76; Ortec, Ametek-AMT, USA). The detector featured an operating energy range of 59–3,200 keV. Its relative efficiency compared to a standard NaI(Tl) detector was 40%, with a full width at half maximum (FWHM) resolution of 1.85 keV at the 1.33 MeV line of ^60^Co, and a peak-to-Compton ratio of 64:1. Digital signal processing was managed via a multichannel analyzer (model DSPEC 502; Ortec, Ametek-AMT, USA). Energy and efficiency calibrations were performed using a certified multi-nuclide gamma standard source embedded in an epoxy resin matrix (density: 1.5 g/cm^3^), covering an energy range from 59 to 1,330 keV (source no. BW/Z-62/73/24; POLATOM, Poland). The counting time for each sample was 172,800 s, whereas the instrument background was determined through a minimum acquisition time of 10 days. Measurement uncertainties were reported at the 1-sigma level (two standard deviation). Spectral analysis and data processing were executed using Maestro-PRO (v9.00.02) and GammaVision (v9) software packages (Ortec-Ametek, USA).

The specific activities of ^137^Cs and ^40^ K were calculated directly from the net peak areas at their respective photopeaks of 661.66 keV and 1460.83 keV. Due to the direct spectral interference between the primary gamma-ray emission of ^226^Ra (186.21 keV) and ^235^U (185.72 keV), the quantification of ^226^Ra was performed indirectly through its decay progeny. The radioactive decay process of ^226^Ra proceeds via alpha emission to gaseous ^222^Rn, which subsequently decays through a series of short-lived radionuclide intermediates, including ^214^Bi and ^214^Pb before reaching stable ^206^Pb. Once the secular radioactive equilibrium in the sealed Marinelli beakers is established, the activities of ^214^Pb and ^214^Bi become equal to that of the parent ^226^Ra. Consequently, the specific activity of ^226^Ra was calculated as the arithmetic mean of the activities derived from its equilibrated progeny: ^214^Bi (609.31 keV and 1764.50 keV) and ^214^Pb (295.22 keV and 351.93 keV). A similar approach was applied to ^232^Th, its specific activity was determined assuming secular equilibrium with its decay products, based on the photopeaks of ^212^Pb (238.63 keV), ^208^Tl (583.19 keV), and ^228^Ac (911.20 keV).

The specific activities of the above-mentioned radionuclides served as the baseline data for evaluating the radiological risk indices of the investigated soils. The mathematical equations utilized to calculate the individual radiological parameters are summarised in Table 2.

**Table 2:** Radiological risk assessment indices

Parameter	Unit	Equation
Radium Equivalent Activity, Ra_eq_	Bq kg^−1^	${Ra}_{eq}={A}_{Ra}+{\mathrm{1,43}\bullet A}_{Th}+\mathrm{0,0077}\bullet {A}_{K}$
		Where: A_Ra_, A_Th_, A_K_ –the specific activity of Ra-226, Th-232 and K-40 [Bq kg^−1^]
		The recommended value should not exceed 370 Bq kg^−1^, which corresponds to an effective dose of 1.5 mGy year^−1^ resulting from exposure to gamma radiation (Szaciłowski, 2024)
Gamma Level Index, Iγ	Unitless	${\mathrm{I}}_{\upgamma }=\frac{{A}_{Ra}}{150}+\frac{{A}_{Th}}{100}+\frac{{A}_{K}}{1500}$
		Where: A_Ra_, A_Th_, A_K_ –the specific activity of Ra-226, Th-232 and K-40 [Bq kg^−1^]
		It refers to the presence of radionuclides classified as NORM, particularly in the surface layer of the material (Zakaly et al. 2024)
Annual Gonadal Dose Equivalent, AGDE	μSv year^−1^	$AGDE=3.09{A}_{Ra}+4.18{A}_{Th}+0.314{A}_{K}$
		Where: A_Ra_, A_Th_, A_K_ –the specific activity of Ra-226, Th-232 and K-40 [Bq kg^−1^]
		AGDE is defined as a measure of the genetic significance of the annual dose of ionising radiation received by the reproductive organs of people living in a given area. The global average is 300 μSv year^−1^ (UNSCEAR, 2000)
Excess Lifetime Cancer Risk, ELCR	Unitless	$ELCT={D}_{out}\bullet T\bullet RF$
		$${D}_{out}={D}_{R}\bullet 8760 \bullet \mathrm{0,2}\bullet \mathrm{0,7}\bullet {10}^{-6}$$
		$${D}_{R}=\mathrm{0,462}\bullet {A}_{Ra}+\mathrm{0,604}\bullet {A}_{Th}+\mathrm{0,0417}\bullet {A}_{K}+\mathrm{0,03}\bullet {A}_{Cs}$$
		Where: A_Ra_, A_Th_, A_K_ –the specific activity of Ra-226, Th-232 and K-40 [Bq kg^−1^]; D_OUT_ – equivalent effective annual dose received outdoors [mSv year^−1^], T – life expectancy (70 years, according to WHO data from 2014), RF – cancer risk factor (0.057 per Sv for stochastic effects of ionising radiation as proposed by the ICRP), D_R_ – gamma radiation dose rate [nGy h^−1^]. The factor 0.2 refers to the estimated amount of time spent outdoors. The conversion factor (0.7 Sv Gy^−1^) is used to convert the absorbed dose at a height of 1 m above ground level into the effective dose received by an adult. The value 8760 represents one year expressed in hours, and 10⁻⁶ is the conversion factor used to convert a dose expressed in nGy to mSv
		The ELCR refers directly to the effect of ionising radiation on cancer risk. It is used to calculate the lifetime probability of the development of cancerous lesions over an individual's lifetime in instances where exposure to gamma radiation from radionuclides present in the soil has occurred (UNSCEAR, 2000), (Bulubasa et al., 2021)

### Elemental analysis

The elemental composition of soil samples was analysed using an energy-dispersive X-ray fluorescence spectrometer (Epsilon 5 ED-XRF, PANalytical, The Netherlands). The method enabled the quantitative analysis of major and trace elements, including metals. Sample preparation and instrumental calibration were carried out according to the manufacturer’s instructions.

### Pollution indices

To assess the extent of soil contamination and the anthropogenic contribution of metals, the geoaccumulation index (Igeo) and enrichment factor (EF) were calculated. The Igeo was calculated using the equation from Müller (1969, 1981).

$$Igeo={log}_{2}\left(\frac{Cn}{1.5\times Bn}\right)$$

where Cn—the measured concentration of the element in soil, and Bn—the geochemical background value. The factor 1.5 accounts for natural variability in background concentrations.

The enrichment factor (EF) was calculated as the ratio of the metal concentration in contaminated soil to its background level:

$$EF=\frac{Csample}{Cbackground}$$

Control soils collected near each crater served as background values. EF values were interpreted as follows: < 2 (minimal enrichment), 2–5 (moderate), 5–20 (significant), and > 20 (very high enrichment).

### Soil pH Measurement

Soil pH was determined following the method described by Thomas (1996). Measurements were performed using a Knick Portavo 904® pH meter (Knick, Germany). Soil suspensions were prepared under controlled laboratory conditions, and pH values were recorded after the electrode reading had stabilised.

### Particle Size Distribution and Soil Texture

Particle size distribution was measured by laser diffraction using a Mastersizer 3000 analyser (Malvern Instruments, UK). Soil samples were dispersed in deionised water with sodium hexametaphosphate (0.05%) to prevent aggregation. The mean particle diameter (μm) was calculated as a volume-weighted average.

### Plant Material and Experimental Design.

Winter wheat plants of the cv. ‘Podolyanka’, grown in Ukraine's Forest-Steppe and Polissya regions, served as the experimental material. The variety ‘Podolyanka’ is noted for its high winter hardiness, drought tolerance, substantial yield potential, and adaptability across various environmental conditions. This variety is among the most widely cultivated intensive-type winter bread wheat varieties in Ukraine, with a potential yield of 11.0–12.0 t/ha (Ministry of Economy of Ukraine, 2026). The effects of lead contamination on morphophysiological parameters and root anatomical structure were investigated in 14-day-old wheat plants. Seeds were washed with water, surface-sterilised in 70% ethanol for 3 min, and rinsed with distilled water. They were then placed in cuvettes containing distilled water and incubated in a thermostat at + 20 °C for 24 h. Germinated seeds were subsequently grown in a hydroponic system containing Knop's medium supplemented with Pb(NO₃)₂ at final concentrations of 0.005, 0.025, 0.05, 0.075, and 0.5 mM (Pb^2+^). The selected Pb concentrations (0.005–0.5 mM Pb(NO₃)₂) were chosen to cover a broad gradient of Pb exposure, including low, moderate, and high stress levels commonly employed in hydroponic studies of wheat and other crop species, allowing the evaluation of dose-dependent physiological responses (Lamhamdi et al., 2013; Kumar & Misra, 2023). The containers with seeds were incubated in the thermostat for a further 24 h and then transferred to a climate chamber (VS3 7100, Vötsch GmbH, Balingen, Germany), where plants were cultivated under controlled conditions at 22 °C, a photosynthetic photon flux density of 160 μmol m⁻^2^ s⁻^1^, a 16/8 h (light/dark) photoperiod, and a relative humidity of 65 ± 5% until day 14 of growth. Morphophysiological parameters were recorded on day 14 of the vegetation period. Control plants were grown in Knop's medium without Pb. Both shoots and roots were collected for further analysis.

### Estimation of Germination and Growth Measurements

The Stress Tolerance Indices (STI, %) for different growth parameters were determined according to Wilkins (1957) using the following equation:

$$STI=\left(\frac{Vstress}{Vcontrol}\right)\times 100\%$$

where V_stress_ and V_contro_ represent the values of the parameter under lead stress and control conditions, respectively. The indices included Root and Shoot Length Stress Tolerance Indices (RLSTI, SLSTI), Root and Shoot Fresh Weight Stress Tolerance Indices (RFSTI, SFSTI), and Root and Shoot Dry Weight Stress Tolerance Indices (RDSTI, SDSTI).

The response of roots to lead ion exposure was assessed using the phytotoxicity index (PP, %), calculated according to the following formula:

### $PProot=\frac{DWc-DWm}{DWc}\times 100\%$_._

Where DWc signifies the average dry weight of roots cultivated in a lead-free medium (control, 0), and DWm indicates the average dry weight of roots grown in a medium containing lead (Amin et al., 2013; Paksoy, Acar, 2009).

### Histological analyses

Roots were collected from 14-day-old wheat plants. This stage was chosen due to specific features of root anatomical development. The roots were fixed in FAA solution (formalin: acetic acid:70% ethanol, 5:5:90) at room temperature for 24–48 h. Transverse Sects. (20–50 μm thick) were prepared in the basal zone (5–10 mm below the root-shoot junction) following the method of Baluska et al. (2013). Sections were stained with 1% toluidine blue and mounted on glass slides in 50% glycerol (Abràmoff et al., 2004). Suberin deposition was detected using Sudan III staining (sections incubated at 70 °C for 10 min). Lignin was visualised using phloroglucinol/HCl (Brundrett et al., 1988). Samples were examined using an Olympus BX53 light microscope equipped with Nomarski differential interference contrast (DIC) optics (Olympus Corporation, Tokyo, Japan). Digital images were captured using a digital camera and analysed with AxioVision 4.9 software.

To quantitatively evaluate the anatomical characteristics of the roots, the following indices were determined:

Stele Ratio (SR, %):

$$SR=\frac{Astele}{Atotal} \times 100\%$$

where A_stele_ is the stele area (vascular cylinder) and A_total_ is the root`s total cross-sectional area (Schneider et al., 2004; Battey, 2003).

Cortex Porosity Index (CPI, %):

$$CPI=\frac{Aintercellular}{Acortex } \times 100\%$$

where A_intercellular_ is the area of intercellular spaces and A_cortex_ is the total area of the root cortex (Ratnayaka et al., 2003).

Xylem Area Ratio (XAR, %):

$$XAR=\frac{Axylem}{Astele}\times 100\%$$

where A_xylem_ is the total area of xylem vessel lumens and A_stele_ is the stele area (Tyree & Zimmermann, 2002; Hacke et al., 2001).

Wall Reinforcement Index (WRI):

$$WRI=\frac{Twall}{Dusssel}$$

where T_wall_ is the cell wall thickness and D_vessel_ is the vessel lumen diameter.

This modified index, initially proposed by Hacke et al. (2001), serves as an indicator of xylem resistance to implosion and as a measure of the hydraulic safety of the conducting system (Tyree & Zimmermann, 2002; Piermattei et al., 2020).

### Statistical analyses

Statistical analyses were performed using Statistica 10.0 (StatSoft Inc., Tulsa, OK, USA). Soil data are presented as the mean ± standard deviation (SD) of five analytical replicates obtained from each homogenized composite soil sample.

For the hydroponic experiment, data are presented as mean ± SD. Differences among Pb treatments were evaluated using one-way analysis of variance (ANOVA), followed by Tukey's honestly significant difference (HSD) test for pairwise comparisons. Statistical significance was accepted at P < 0.05. The effect size (η^2^) was calculated to estimate the proportion of total variance explained by Pb concentration. Pearson's correlation coefficients (r), coefficients of determination (r^2^), and 95% confidence intervals were calculated to evaluate the relationships between Pb concentration and the measured plant traits.

## Results

### Analysis of Soil Physicochemical Properties

Analysis of samples indicated that the soils in the studied area possess a typical aluminosilicate composition with high quartz content (SiO₂ 35–45%, Al₂O₃ 9–13%, CaO 0.8–7.7%, Fe₂O₃ 1.4–4%). Additionally, the levels of metals at missile impact points (samples marked with a stroke) were significantly elevated compared to background levels in nearby control plots, suggesting localised contamination directly associated with missile strike sites (Table 3).

**Table 3:** Concentrations of metals in control and missile-affected soil samples (mg kg⁻^1^dry weight)

Metal	1	1′	2′	3	3′	4	4′	5	5′	6	6′	7	7′
Cr	122.0 ± 4.5	961.7 ± 31.2	535.7 ± 3.2	80.7 ± 2.0	963.3 ± 3.1	73.0 ± 3.6	987.0 ± 3.6	72.3 ± 1.2	626.3 ± 3.2	76.3 ± 1.5	977.7 ± 1.5	178.0 ± 1.2	836.7 ± 11.5
Mn	333.0 ± 2.6	702.0 ± 2.6	2782.0 ± 2.0	340.3 ± 1.5	1083.0 ± 3.0	193.0 ± 4.4	1192.0 ± 1.6	290.0 ± 1.4	1091.0 ± 1.0	443.0 ± 2.6	1214.0 ± 2.6	483.0 ± 2.6	1101.7 ± 2.9
Ni	21.0 ± 1.7	141.3 ± 3.1	199.7 ± 0.6	31.3 ± 1.2	20.3 ± 0.6	12.7 ± 2.5	187.0 ± 11.3	10.0 ± 1.0	155.3 ± 2.3	20.0 ± 1.0	160.3 ± 0.6	31.7 ± 2.9	134.7 ± 1.2
Cu	51.7 ± 1.5	203.7 ± 5.8	382.3 ± 3.1	50.3 ± 0.6	380.7 ± 0.6	45.0 ± 3.0	202.0 ± 0.0	31.3 ± 3.2	241.3 ± 2.3	43.3 ± 2.9	230.0 ± 2.9	62.0 ± 3.5	189.7 ± 0.6
Zn	90.3 ± 0.6	250.0 ± 1.0	321.7 ± 2.9	81.0 ± 1.7	907.0 ± 1.7	102.3 ± 3.2	560.0 ± 0.0	51.0 ± 1.0	672.0 ± 2.1	41.3 ± 1.2	771.3 ± 3.1	72.0 ± 3.5	281.3 ± 2.3
Pb	21.7 ± 2.9	150.3 ± 0.6	243.0 ± 4.2	21.3 ± 2.3	231.3 ± 1.5	22.0 ± 2.6	198.0 ± 0.0	30.3 ± 0.6	171.7 ± 2.9	17.0 ± 6.1	205.0 ± 4.6	32.0 ± 3.5	175.0 ± 4.0

Sites marked with a prime symbol (′) represent missile impact locations (explosion craters), whereas sites without the prime denote the corresponding control plots. Site 1 served as the common control for both impact sites (Sites 1′ and 2′); therefore, a separate control site (Site 2) was not established. Values are presented as mean ± standard deviation (SD) of five analytical replicates obtained from homogenized composite soil samples (*n* = 5)

The concentration of Cr ranged from 70 to 980 mg/kg. Significant exceedances of the background level (< 100 mg/kg, according to literature data) were observed at points 1′, 2′, 3′, 4′, and 6′. Mn content varied from1192 to 2782 mg kg⁻^1^, with the highest values (> 1000 mg/kg) recorded at points 2′, 4′, 5′, and 6′. Ni ranged from 141.3 to 187 mg kg⁻^1^, exceeding the background level (20 mg/kg) at points 2′, 4′, and 6′. Cu reached 382 mg kg⁻^1^ at point 2′, more than five times the background value of approximately 50 mg/kg. The maximum concentrations of Zn and Pb were 907 mg kg⁻^1^ (at point 3′) and 243 mg kg⁻^1^ (at point 2′), respectively. Control plots showed concentrations close to natural background levels, confirming the local origin of the contamination. Table 4 compares metal concentrations in soils from explosion craters with local background values, literature ranges, and regulatory limits. The data indicate significant enrichment of several elements in impacted soils. The highest exceedances were recorded for Cr (up to 987 mg kg⁻^1^), Cu (382 mg kg⁻^1^), Zn (907 mg kg⁻^1^), and Pb (243 mg kg⁻^1^), greatly surpassing both natural background levels and permissible concentrations. Nickel also showed elevated values in selected samples. In contrast, control soils exhibited concentrations close to typical geochemical background ranges, confirming the localised nature of contamination. The observed pattern suggests technogenic metal input associated with explosive events and supports the selection of lead as a representative toxicant for plant response analysis. Regarding the sources of contamination, detailed information on the composition of structural elements and warhead components of modern missiles is limited in open sources. Nevertheless, the increased concentrations of metals in soils near explosion sites are a predictable consequence of ammunition detonation. Metals are released from the missile casing, engine, and warhead due to destruction and combustion, and the explosion products, along with fine particulate matter, settle in the upper soil layer. A similar pattern of local metal accumulation has been described in numerous studies on the impact of military activities on soils (Broomandi et al., 2020; Hlavatskyi et al., 2024Solokha et al., 2024).

**Table 4:** Summary comparison of metal concentrations in soils with local background values, literature ranges, and maximum allowable concentrations (MAC) (mg kg⁻^1^dry weight)

Metal	Local background (control soils), mg kg⁻^1^	Literature range (mg kg⁻^1^)	Regulatory threshold (MAC), mg kg⁻^1^	Maximum detected concentration (mg kg⁻^1^)
Cr	70–170	20–100	100	987 (sample 4′)
Mn	190–480	300–1000	1500	2782 (sample 2′)
Ni	10–30	10–40	40	200 (sample 2′)
Cu	30–60	20–50	55	382 (sample 2′)
Zn	40–100	50–100	220	907 (sample 3′)
Pb	20–30	10–30	32	243 (sample 2′)

Samples marked with a prime symbol (′) represent missile impact locations. Local background values were determined from control soils collected within the same study area. Literature concentration ranges were compiled from Kabata-Pendias (2011) and Tóth et al. (2016). Maximum allowable concentrations (MAC) are based on Ukrainian soil quality standards and international environmental guidelines (European Commission, 2012; USEPA, 2023)

The calculated pollution indices confirmed substantial anthropogenic enrichment of Pb in crater soils. The enrichment factor (EF) ranged from 6.5 to 10.4, indicating significant enrichment relative to background levels. The geoaccumulation index (Igeo) ranged from 2.12 to 2.80, corresponding to the “moderately to strongly contaminated” category. These results demonstrate that lead accumulation is primarily associated with technogenic input rather than natural geochemical variability The variability of Pb concentrations across crater sites was moderate (CV ≈ 16%), indicating relatively consistent contamination levels, a pattern further supported by low variation in Igeo values (CV ≈ 9%) (Table 5).

**Table 5:** Lead concentrations, enrichment factor (EF), geoaccumulation index (Igeo), and contamination classification for missile-affected soils

Site	Pb (mg kg^−1^)	Background (mg kg^−1^)	EF	Igeo	Contamination class
1′	150	22	6.5	2.12	Moderately–strongly contaminated
2′	243	22	10.4	2.80	Moderately–strongly contaminated
3′	231	21	10.0	2.74	Moderately–strongly contaminated
4′	198	22	8.6	2.52	Moderately–strongly contaminated
5′	172	30	7.4	2.30	Moderately–strongly contaminated
6′	205	17	8.7	2.54	Moderately–strongly contaminated
7′	175	32	7.4	2.30	Moderately–strongly contaminated

Background Pb concentrations correspond to the associated control plots. The enrichment factor (EF) and geoaccumulation index (Igeo) were calculated using the corresponding local background concentrations. Contamination classes were assigned according to the Igeo classification proposed by Müller (1969, 1981): Class 0 (Igeo ≤ 0), uncontaminated; Class 1 (0 < Igeo ≤ 1), uncontaminated to moderately contaminated; Class 2 (1 < Igeo ≤ 2), moderately contaminated; Class 3 (2 < Igeo ≤ 3), moderately to strongly contaminated; Class 4 (3 < Igeo ≤ 4), strongly contaminated; Class 5 (4 < Igeo ≤ 5), strongly to extremely contaminated; Class 6 (Igeo > 5), extremely contaminated

Thus, based on an analysis of concentrations relative to local background levels and regulatory standards, a significant exceedance of metal content has been identified in samples collected from craters. A less pronounced but consistent exceedance is observed for Cu and Ni, whereas Mn shows greater variability and is likely less specific to explosive impact. The most substantial increases are recorded for Pb, Zn, and Cr, whose concentrations exceed both local background levels and permissible limits by several times. These elements can therefore be considered key indicators of technogenic impact associated with explosions.

### pH and granulometric composition of the soil.

Soil pH analysis revealed a pronounced decrease in acid–base parameters in explosion craters compared with control sites. The most pronounced changes were recorded at sites 3′ (pH 7.76 vs. 8.73 in the control), 4′ (7.18 vs. 9.35), and 7′ (7.21 vs. 7.60), indicating substantial local acidification of the soil environment within zones subjected to intense mechanical impact (Table 1). In contrast, at some investigated locations (notably 1′: pH 8.91 and 5′: pH 8.89), a slight increase in alkalinity was observed, suggesting spatial heterogeneity in soil chemical transformation processes and a possible role for the local substrate’s buffering capacity (Table 1). Soil pH is considered one of the most important factors determining the concentration of metals in the soil solution, their mobility, and their availability to plants (Fijalkowski et.al., 2012). A lower (acidic) pH promotes metal dissolution, thereby increasing their mobility and bioavailability.

Granulometric analysis revealed systematic changes in the physical structure of soils across all investigated craters. In most cases, the mean particle size exceeded the corresponding control values, indicating mechanical compaction and the redistribution of soil material under the blast shock wave. The increase in mean particle diameter can be interpreted as resulting from the disruption of the natural soil aggregate structure, followed by the coagulation of fine-grained fractions or the incorporation of coarser clastic components. These changes are accompanied by an increase in soil bulk density, which reduces water permeability, impairs aeration, and restricts gas diffusion within the soil profile.

The comprehensive transformation of soil chemical and physical properties in areas affected by explosions is a critical driver of soil ecosystem degradation. Disruption of the acid–base balance and compaction of the soil profile create unfavourable conditions for vegetation development and suppress the functional activity of the soil microbiota, consistent with the findings of Yu et al. (2017) and Mystrioti & Papassiopi (2024). The results indicate the long-term nature of explosive impacts and their potentially persistent influence on the physicochemical state of soils in affected ecosystems.

The results confirm that explosive loading induces a complex transformation of the soil's physicochemical properties, which persists for at least several years after the impact.

### Gamma – ray spectrometry results

**Fig. 1:**
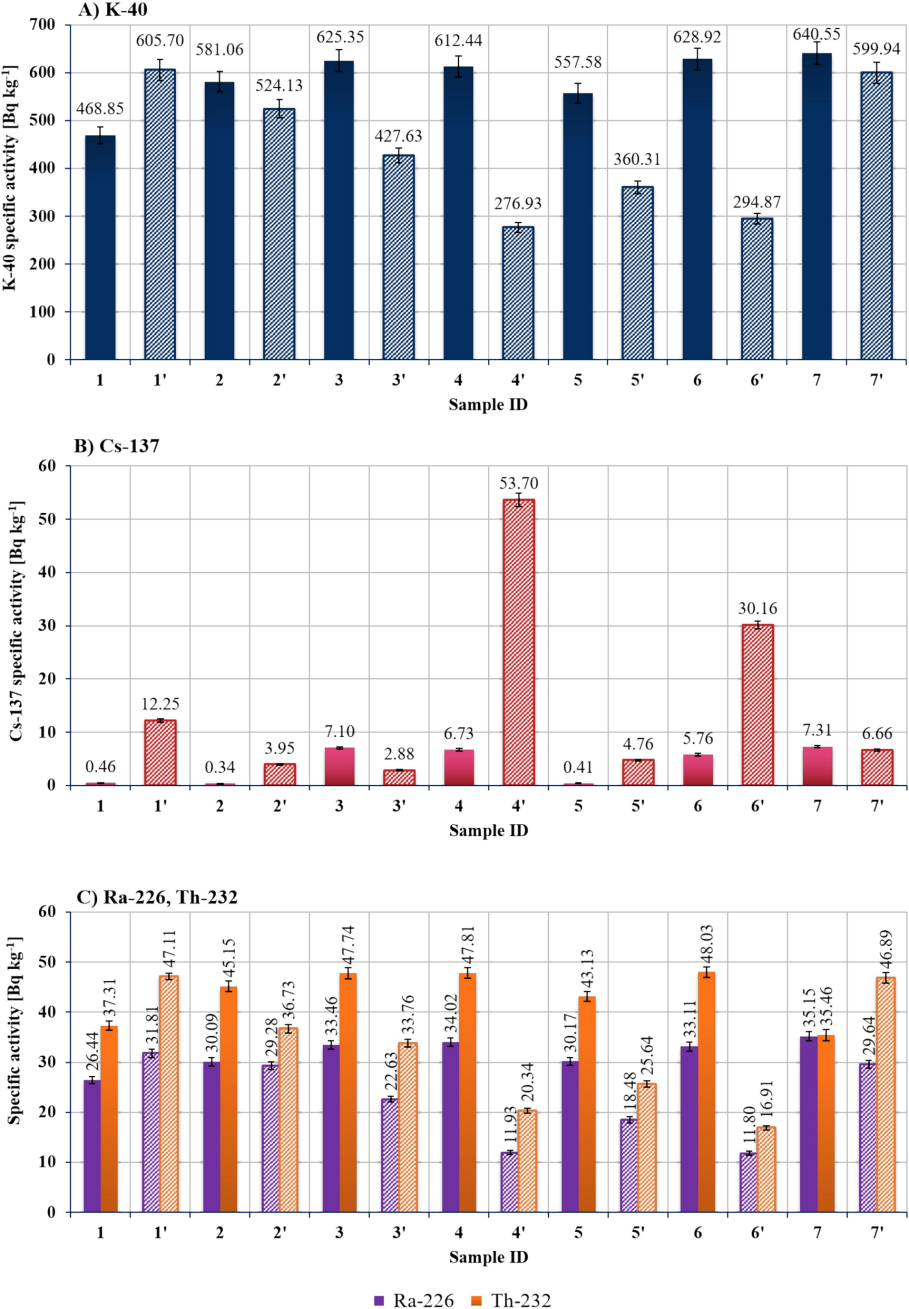
Gamma – ray emitters content in the soil samples. *Note*. Data are presented as mean ± standard error (x ± SE). In accordance with standard nuclear metrology and radiation measurement protocols, these error bars represent the total propagated measurement uncertainty at the 1-sigma level

The results of the gamma-ray emitters content in the soil samples are presented in Fig. 1. As demonstrated, the highest specific activity of K-40 was recorded for sample 7 (640.6 ± 23.26 Bq kq^−1^), in contrast to sample 4', in which the content of naturally occurring radioactive potassium was only 276.9 ± 10.27 Bq kq^−1^. The absence of any discernible disparities between the material collected from the impact craters and the control samples can be attributed to local variations in the K-40 content of the soil. In the case of Ra-226, it was found that sample 6’ exhibited the lowest measured concentration (11.80 ± 0.41 Bq kq^−1^), whilst the highest radioactivity was recorded in sample 7 (35.15 ± 0.91 Bq kq^−1^). A comprehensive analysis of the gamma-ray spectra lead to the conclusion that all of the soils had a higher Th-232 content compared to Ra-226. The maximum level of Th-232 was identified in sample 6 (48.03 ± 1.08 Bq kq^−1^), while the minimum specific activity of this radionuclide was determined in sample 6' (16.91 ± 0.44 Bq kq^−1^). As with K-40, the variations in the specific activity of both Ra-226 and Th-232 in the analysed soil samples are the result of local variations in soil composition, particularly the parent rock, and past human activity. It should be noted that the detonation of an explosive charge exposes deeper layers of soil, which differ from topsoil in terms of composition as well as the dynamics of geochemical and biochemical processes. Consequently, the presence of the Th-232 and Ra-226 compounds in the upper layer of the soil profile was displaced by the detonation, resulting in the exposure of a layer with a lower concentration of these radionuclides. Nevertheless, the activity value parameters ascertained for naturally occurring radioactive substances are consistent with the results reported by other researchers (Menshikova et al., 2021).

Interestingly, the specific activity of the Cs-137 resulting from anthropogenic sources exhibited a considerable range, varying from 0.34 Bq kq^−1^ (sample 2) to 53.70 Bq kq^−1^ (sample 4’). It is noteworthy that the specific activity of Cs-137 in the control samples (1–7) is comparable to the mean concentration of this radionuclide in soils (Popovych et al., 2022). In contrast, in samples collected from the craters left by missile explosions (1', 2', 4', 5', 6'), the level of Cs-137 is significantly higher than in the control samples. The underlying cause may be attributed to the exposure of layers comprising elevated concentrations of radioactive caesium, which have been accumulated in the soil. However, vertical migration of Cs-137 in soil is a very slow process, with most of the released Cs-137 being retained in the topsoil (up to 30 cm) (Kaissas et al., 2023). It can therefore be concluded that a significant contribution of the total Cs-137 specific activity recorded for these samples is attributable to soil contamination by substances present in the explosive material/projectile components.

### Radiological risk indices

The radiological hazard indices calculated for the analyzed soil samples are summarized in Table 6. These parameters assess exclusively Naturally Occurring Radioactive Materials (NORM) and omit the anthropogenic contribution of ^137^Cs.

**Table 6:** Radiological hazard indices calculated for the analyzed soil samples

	Radiological hazard indices			
Sample ID	Ra_eq_, Bq kg^−1^	Iγ	AGDE, μSv year^−1^	ELCR ∙ 10^–3^
1	115.90	0.86	384.90	0.27
1′	145.81	1.09	485.37	0.34
2	139.40	1.04	464.15	0.32
2′	122.15	0.91	408.56	0.28
3	149.88	1.12	499.31	0.35
3′	103.84	0.77	345.33	0.24
4	149.54	1.11	497.26	0.34
4′	62.34	0.47	208.83	0.15
5	134.78	1.00	448.59	0.31
5′	82.90	0.62	277.43	0.19
6	150.21	1.12	500.53	0.35
6′	58.68	0.44	199.72	0.14
7	135.17	1.02	457.95	0.32
7′	142.89	1.07	475.98	0.33
Recommended safety threshold (UNSCEAR, 2000)	370.00	1.00	1,000	0.29

The radium equivalent activity (Ra_eq_) values ranged from 58.68 Bq kg^−1^ to 150.21 Bq kg^−1^. For all analyzed samples, Ra_eq_ remained consistently below the internationally recommended maximum threshold of 370 Bq kg^−1^. Gamma Level Index (Iγ) varied between 0.44 and 1.12, with the upper safety limit exceeded in 8 out of 14 samples (57%). Given that Iγ refers to the gamma radiation flux originating from the surface soil layer, these data provide essential baseline criteria for evaluating land suitability for potential agricultural or residential development. The health-related radiological risks were evaluated using the Excess Lifetime Cancer Risk (ELCR) and the Annual Gonadal Dose Equivalent (AGDE). The former estimates the probability of radiation-induced carcinogenesis over a lifespan, whereas AGDE serves as an indicator of potential genetic damage to reproductive organs. According to the United Nations Scientific Committee on the Effects of Atomic Radiation guidelines (UNSCEAR, 2000), the global average background AGDE value is 300 μSv year^−1^. In this study, the calculated AGDE values ranged from199.72 to 500.53 μSv year^−1^. Taking into account the established threshold value (at which point risk of damage to germ cells and radiation-sensitive organs begins to increase), the direct contact with any of the soil samples will not lead to an increase in human radiation exposure. The results between 300 and 1,000 μSv year^−1^ indicate elevated local gamma radiation levels relative to the global average. However, they do not present an immediate threat of acute germ cell or tissue damage, remaining within acceptable parameters for the general population. A corresponding trend was observed for the ELCR index, ranged from 0.19∙10^–3^ to 0.35∙10^–3^, with 8 samples exceeding the standard safety threshold. Nevertheless, synthesizing these indices with the overall NORM activity concentrations suggests that long-term exposure to the baseline gamma radiation of the investigated soils does not indicate a statistically significant elevation in oncogenic risk.

To evaluate the statistical significance of the differences in radiological risk parameters between the impact craters and their respective control locations, a two-tailed Wilcoxon signed-rank test for paired samples was performed (α = 0.05, n = 7). For the radium equivalent activity, the smaller sum of ranks was calculated as W = 4, which exceeds the critical value of W_crit_ = 2. Consequently, the observed downward trend in radiological risks within the impact craters does not demonstrate a statistically significant deviation from the control background (p > 0.05). This statistical outcome is primarily driven by the localized geochemical anomalies observed in sample pairs 1–1’ and 7–7’, where the crater values paradoxically exceeded the control levels. These results imply that while energetic displacement of soil during detonation generally promotes the exposure of deeper, less active lithological layers, local post-blast heterogeneity and specific geological features can override this general trend.

Furthermore, a correlation analysis (e.g., Pearson’s or Spearman’s) between the calculated radiological risk indices (such as Raeq, Iγ, etc.) with the specific activities of their constituent radionuclides (e.g., Ra-226) was intentionally omitted. Since these indices are derived directly from rigid mathematical equations where radionuclide activities function as internal independent variables, any correlation analysis would inherently yield artificially inflated, tautological coefficients. Consequently, such results would reflect a spurious statistical relationship rather than a genuine, independent geochemical correlation.

Analysis of the radiometric data indicated that the lowest values for all calculated radiological risk parameters occurred in sample 6', whereas the highest values were recorded in the soil designated as 6 (the control sample for 6'). Notably, the radiological risk parameters are lower for samples obtained from impact craters in comparison to the control samples (with the exception of samples 1’ and 7’). This phenomenon may be attributed to the displacement of soil material during the detonation events; the release of substantial kinetic energy likely caused the excavation and exposure of deeper, less active lithological layers. Furthermore, subsequent environmental processes, including weathering and erosion, may have enhanced the depletion or redistribution of gamma-ray emitting radionuclides within the craters, directly influencing the calculated parameters. Overall, the evaluated indices demonstrate the absence of an immediate or significant radiological hazard to human health. The observed elevations in specific parameters are primarily governed by the localized geogenic background variations of K-40 and Th-232, remaining well within the globally accepted safe thresholds for the general population.

### Influence on the morphophysiological parameters of 14-day-old *T. aestivum* seedlings

**Fig. 2:**
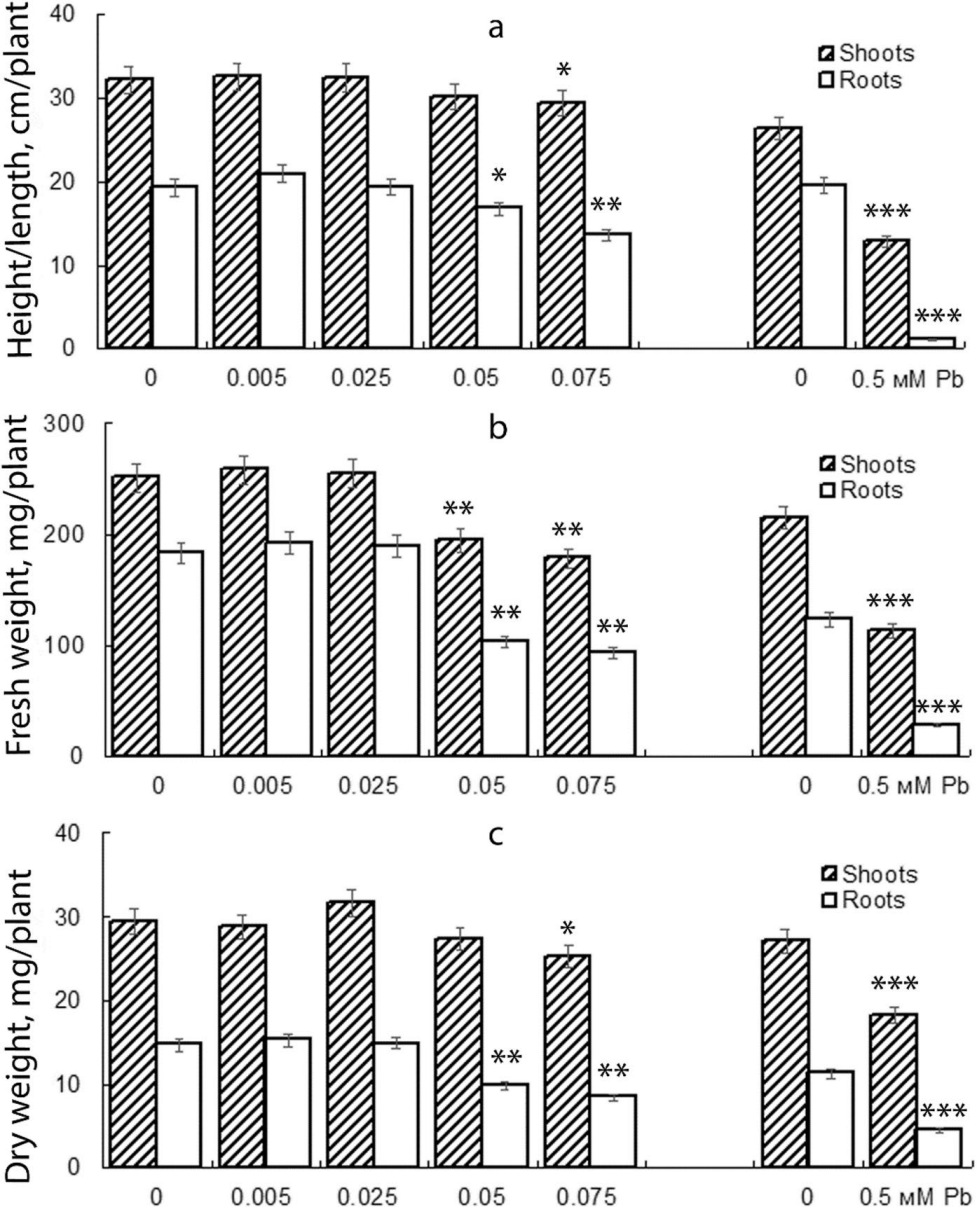
The effect of lead on the morphophysiological parameters of the shoots and roots of 14-day-old *Triticum aestivum* L. cv. ‘Podolyanka’ plants. Notes: Means followed indicate a significance at * – P < 0.05. ** – P < 0.01 and *** – P < 0.001 compared to the control (0 Pb^2+^) according to the Tukey test. n = 90 pcs; x ± standard error (SE)

Prolonged exposure of 14-day-old plants to lead-containing solutions at 0.005 and 0.025 mM did not affect shoot height or root length. In contrast, growth in solutions containing 0.05, 0.075, and 0.5 mM Pb reduced shoot height by 6.5%, 8.7%, and 51.5%, and root length by 13.1%, 29.5%, and 93.8%, respectively (Fig. 2A).

Changes in FW accumulation were also concentration-dependent. At 0.005 and 0.025 mM Pb, FW in both shoots and roots did not differ significantly from the control. However, at 0.5 mM Pb, shoot and root FW decreased to 113.4 ± 5.7 and 28.4 ± 1.4 mg per plant, respectively, representing reductions of 47.3% and 77.1% compared with the control (Fig. 2B).

Shoot DW showed a gradual decline from 0.025 mM Pb. At this concentration, DW was 7.8% higher than the control, whereas at 0.05, 0.075, and 0.5 mM, it was reduced by 6.8%, 13.9%, and 32.5%, respectively. A significant decrease in root DW was observed at Pb concentrations of 0.05–0.5 mM, with reductions of 33.3%, 48.5%, and 60.2%, respectively (Fig. 2C).

One-way ANOVA showed that lead concentration had a statistically significant effect (p < 0.001) on all investigated morphophysiological parameters, as indicated by high η^2^ values (up to 98.6%). The strongest effects were observed for root FW (F(4,45) = 789.07, p < 0.001), root DW (F(4,45) = 507.13, p < 0.001), and shoot FW (F(4,45) = 217.48, p < 0.001), indicating high sensitivity of the root system to metal toxicity. The η^2^ values indicate that lead concentration accounts for 60.5% of the variation in shoot height and up to 98.6% of the variation in root FW. Overall, the ANOVA results confirm a statistically significant effect of Pb^2+^ concentration on all measured parameters in 14-day-old plants.

Correlation analysis results (Table 7) between Pb concentration and morphophysiological traits revealed strong negative relationships, with correlation coefficients (r) ranging from − 0.92 to − 0.94 (p < 0.05) for most parameters. An exception was shoot DW (r =  − 0.73, p = 0.16), which showed lower statistical significance, likely due to the stimulatory effect observed at 0.025 mM Pb (+ 7.8% relative to control). The coefficients of determination (r^2^), ranging from 53.4% (for shoot DW) to 88.8% (for root length), indicate that lead concentration is the primary factor governing variation in morphophysiological responses of 14-day-old plants.

**Table 7:** Pearson correlation coefficients (r) between lead concentration and morphophysiological parameters of 14-day-old seedling *T. aestivum*

Indicators	r	R^2^	p	95% CI
Shootheight	-0.94	87.9	0.017	[-0.996. -0.320]
Rootlength	-0.94	88.8	0.016	[-0.996. -0.358]
Shoot FW	-0.94	88.3	0.017	[-0.996. -0.338]
Root FW	-0.92	84.6	0.027	[-0.995. -0.198]
Shoot DW	-0.73	53.4	0.161	[-0.981. + 0.427]
Root DW	-0.94	88.7	0.017	[-0.996. -0.351]
SLSTI	-0.94	88.0	0.018	[-0.996. -0.318]
RLSTI	-0.94	89.2	0.015	[-0.996. -0.368]
SFSTI	-0.94	88.3	0.018	[-0.996. -0.339]
RFSTI	-0.92	84.7	0.027	[-0.995. -0.200]
SDSTI	-0.73	53.4	0.161	[-0.981. + 0.428]
RDSTI	-0.94	88.6	0.017	[-0.996. -0.350]

95% CI – confidence interval. Correlations are considered statistically significant at p < 0.05

**Fig. 3:**
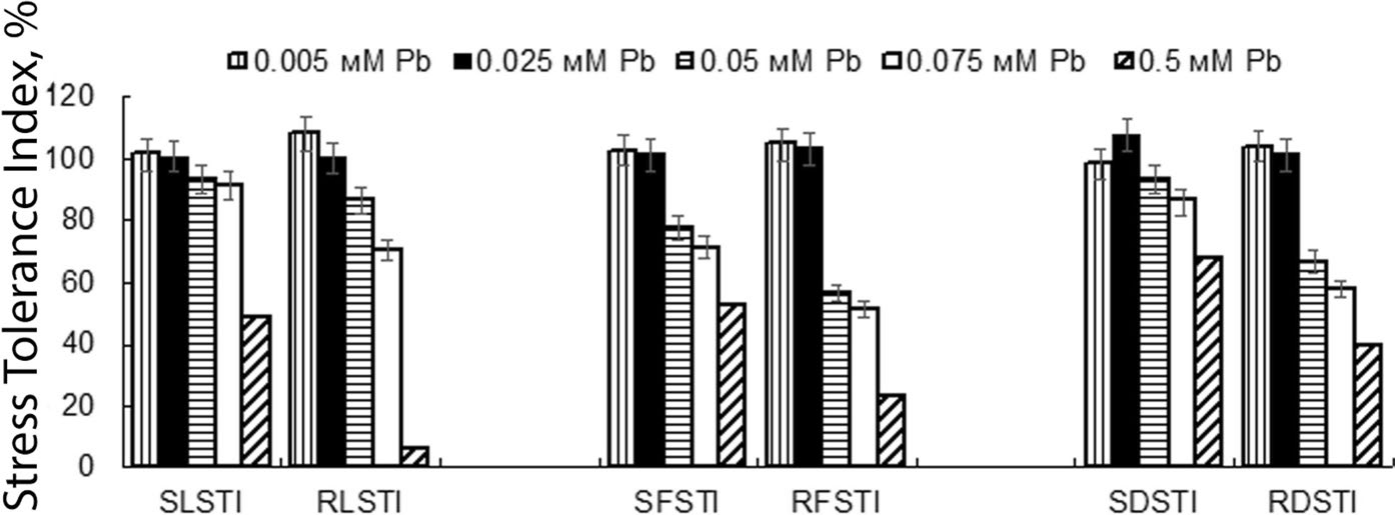
The effect of different lead concentrations on shoot and root Stress Tolerance Indexes (SLSTI, RLSTI, SFSTI, SDSTI, RFSTI, and RDSTI) of 14-day-old seedlings of *T. aestivum.* %

Thus, with increasing stress duration at lead concentrations above 0.05 mM, we observed progressive intensification of negative changes in morphophysiological parameters, particularly in the root system. At the same time, high STI values indicate a stimulatory effect of low lead concentrations (0.005 and 0.025 mM) on the growth of 14-day-old wheat plants (Fig. 3).

Correlation analysis of STI revealed strong, nearly functional inverse relationships between metal concentration and most morphophysiological parameters (r ranging from − 0.92 to − 0.94, p < 0.05), except for SDSTI (r =  − 0.73, p = 0.161), likely due to the nonlinear response of the shoot system to low metal concentrations (Table 8).

**Table 8:** Effect of different lead concentrations on the phytotoxicity index (PP) of root dry weight (DW) in 14-day-old *T. aestivum* seedlings

Lead. mM	PP of root DW, %	Effect of action
0.005	4.1	Weak stimulation
0.025	1.4	Weak stimulation
0.05	33.3	Weak suppression
0.075	42.2	suppression
0.5	60.2	Severe inhibition

**Fig. 4:**
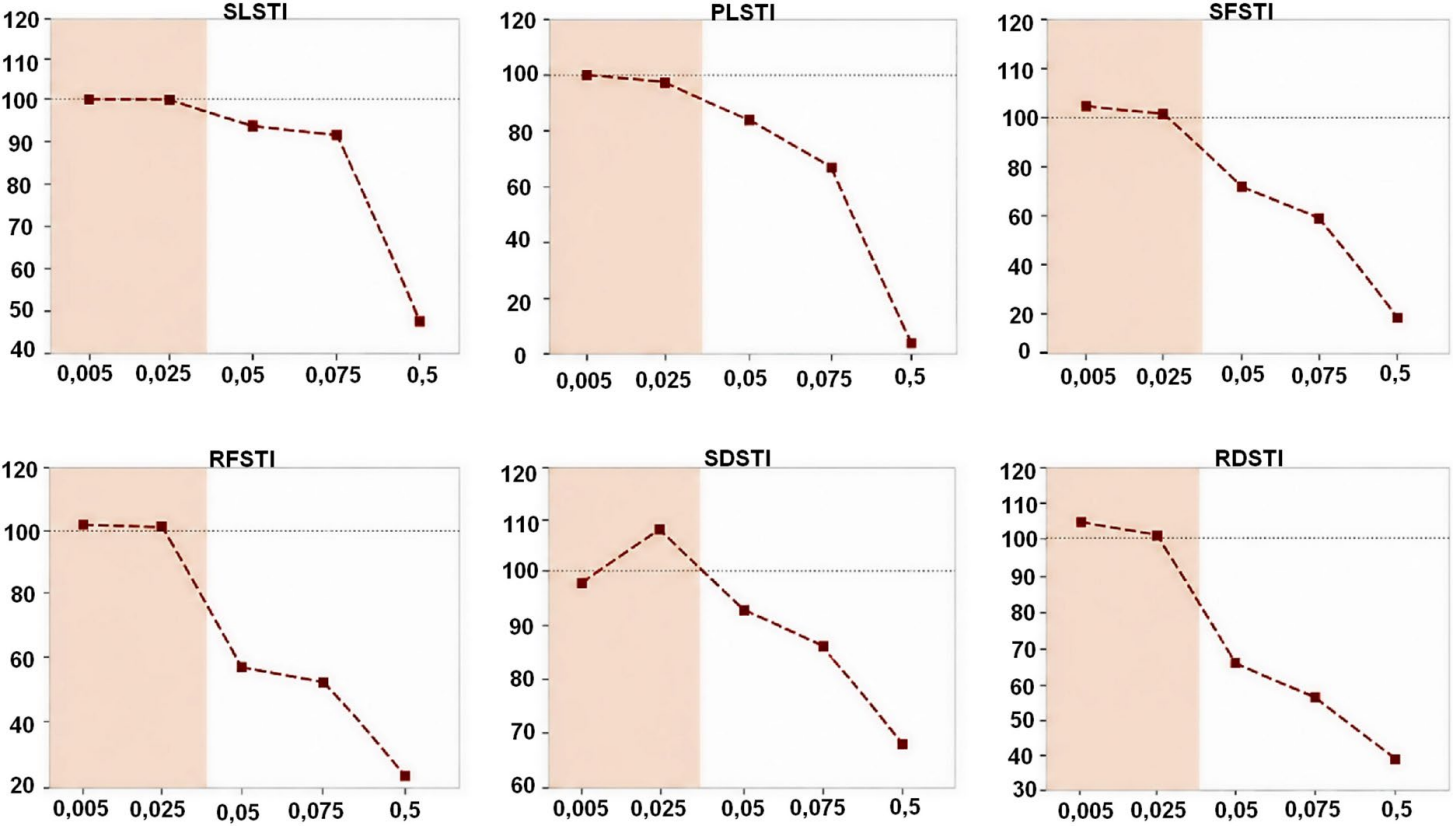
Dynamics of Stress Tolerance Indices (SLSTI, RLSTI, SFSTI, SDSTI, RFSTI and RDSTI) in 14-day-old *T. aestivum* seedlings.Notes: Changes in stress tolerance indices are reported for shoot length (SLSTI), root length (RLSTI), shoot fresh weight (SFSTI), root fresh weight (RFSTI), shoot dry weight (SDSTI), and root dry weight (RDSTI). Values are expressed as percentages relative to the control (STI = 100%). STI values above 100% indicate a stimulatory effect (hormesis)

Analysis of all STI indicators showed a dose-dependent decline in stress tolerance up to 0.5 mM (Fig. 4). The hormetic zone for most STI parameters was observed within the metal concentration range of 0.005–0.025 mM, except for SDSTI.

The PP index is mathematically related to STI and reflects similar trends in the variation of growth parameters, but from a different perspective, namely, the degree of inhibition (PP) versus tolerance (STI). Assessing PP based on root DW serves as an indicator of the primary target of lead toxicity in plants (Mishra &Choudhuri, 1999; Rusan et al., 2015). Our results indicate that lead at concentrations of 0.005 and 0.025 mM exhibited a slight stimulatory effect, whereas at 0.05 and 0.075 mM it exerted a moderate phytotoxic effect. At 0.5 mM, pronounced inhibition of root DW was observed (Table 8).

Thus, lead at concentrations of 0.005 and 0.025 mM did not inhibit but rather stimulated the growth of 14-day-old plants, as evidenced by STI values exceeding 100% and low PP values. The results of the statistical analysis demonstrate a dose-dependent toxic effect of Pb^2^ ions on the morphophysiological parameters of 14-day-old wheat plants. Strong correlations (Pearson’s correlation coefficients ranging from r =  − 0.92 to r =  − 0.94 at p < 0.05) indicate a clear linear relationship between toxicant concentration and plant growth parameters.

### Effect of Lead on the Anatomical Structure of the Roots of 14-day-old *T. aestivum*

To quantitatively assess anatomical changes in wheat roots under Pb exposure, a set of morphometric indices (SR, CPI, XAR, WRI) was used to characterise tissue ratios, cortical porosity, and vascular structural features.

In control plants, the total cross-sectional root area was 22,000 ± 1,500 µm^2^, whereas the stele area was 4,200 ± 300 µm^2^ (19.1 ± 1.2%). Under exposure to 0.05 mM Pb, these parameters did not change significantly (p > 0.05). In contrast, at 0.5 mM Pb, the total cross-sectional area decreased by 15.9%, while the stele area decreased by 36.9%, accompanied by a reduction in SR to 14.3 ± 1.8% (p < 0.05). The epidermal tissues of control plants consisted of a continuous layer of cells with a cell wall thickness of 8.2 ± 0.4 µm. Root hairs measured 1.8 ± 0.2 mm in length and had a density of 12 ± 1.2 hairs/mm. Under exposure to 0.05 mM Pb, cell wall thickness decreased by 14.6% (to 7.0 ± 0.5 µm), while the proportion of deformed cells remained low (5 ± 2%). At 0.5 mM Pb, a pronounced disruption of the epidermal structure was observed: the proportion of deformed cells increased to 25 ± 4%, cell wall thickness decreased to 5.8 ± 0.6 µm, and root hair length and density declined to 0.8 ± 0.3 mm and 5 ± 1.0 hairs/mm, respectively (p < 0.05).

The root cortex of control plants consisted of 8 ± 0.5 cell layers, with a total thickness of 65 ± 4 µm, and intercellular spaces were virtually absent. Under exposure to 0.05 mM Pb^2+^, intercellular spaces began to form (CPI = 8 ± 2%). At 0.5 mM Pb^2+^, cortical thickness decreased to 48 ± 6 µm (p < 0.05), whereas CPI increased to 28 ± 5%, indicating a loss of tissue compactness.

In control plants, the endodermis was at an early developmental stage, characterised by low levels of suberisation (0.5 ± 0.2 points) and lignification (0.3 ± 0.1 points). Upon exposure to 0.05 mM Pb^2+^, these parameters increased to 1.5 ± 0.3 and 1.2 ± 0.2 points, respectively (p < 0.05). At 0.5 mM Pb^2+^, the endodermis progressed to a later developmental stage, with the formation of U-shaped cell wall thickenings; suberisation and lignification levels increased to 4.2 ± 0.4 and 4.5 ± 0.5 points, respectively, while the proportion of deformed cells reached 22 ± 3%.

The vascular system of control plants comprised 4.0 ± 0.3 protoxylem vessels and 5.8 ± 0.4 metaxylem vessels, with an average vessel diameter of 28 ± 2 µm. The total xylem area was 3650 ± 280 µm^2^ (XAR = 86.9%). Under exposure to 0.05 mM Pb^2+^, vessel diameter decreased by 10.7%, vessel wall thickness increased to 2.3 ± 0.2 µm, and XAR declined slightly to 79.4%. At 0.5 mM Pb^2+^, the numbers of protoxylem and metaxylem vessels decreased to 2.5 ± 0.5 and 3.5 ± 0.6, respectively; vessel diameter fell to 18 ± 2.5 µm, and total xylem area declined to 1840 ± 300 µm^2^, reducing XAR to 69.4%. The vessel reinforcement index (WRI) increased from 0.08 (control) to 0.09 and 0.21 at 0.05 and 0.5 mM Pb^2+^, respectively, reflecting increased relative vessel wall thickness alongside reduced vessel diameter.

Thus, morphometric indices confirmed a dose-dependent pattern of root anatomical changes (Table 9). In particular, at a high Pb^2+^ concentration (0.5 mM), a decrease in stele proportion (SR) and xylem area (XAR) was observed, accompanied by increased cortical porosity (CPI) and a higher vessel reinforcement index (WRI). At a low concentration (0.05 mM), the changes were less pronounced and mainly involved an increase in cortical porosity and a slight rise in WRI.

**Table 9:** Lead-induced modifications of root anatomical structure in 14-day-old seedling *T. aestivum*

Parameter	Control	0.05 mM Pb	0.5 mM Pb
Cross-sectional area (µm^2^)	22,000 ± 1,500ᵃ	21,245 ± 1,100ᵃ	18,500 ± 1,176ᵇ
Stele area (µm^2^)	4,200 ± 300ᵃ	4,130 ± 256ᵃ	2,650 ± 400ᵇ
SR (%)	19.1 ± 1.2ᵃ	18.6 ± 1.4ᵃ	14.3 ± 1.8ᵇ
Cell wall thickness of epidermis (µm)	8.2 ± 0.4ᵃ	7.0 ± 0.5ᵇ	5.8 ± 0.6ᶜ
Root hair length (mm)	1.8 ± 0.2ᵃ	1.6 ± 0.3ᵃ	0.8 ± 0.3ᵇ
Root hair density (no/mm)	12 ± 1.2ᵃ	11 ± 1.2ᵃ	5 ± 1.0ᵇ
Cortex thickness (µm)	65 ± 4ᵃ	64 ± 3ᵃ	48 ± 6ᵇ
CPI (%)	not detectedᵃ	8 ± 2ᵇ	28 ± 5ᶜ
Suberization (score)	0.5 ± 0.2ᵃ	1.5 ± 0.3ᵇ	4.2 ± 0.4ᶜ
Lignification (score)	0.3 ± 0.1ᵃ	1.2 ± 0.2ᵇ	4.5 ± 0.5ᶜ
Protoxylem vessels (number)	4.0 ± 0.3ᵃ	4.1 ± 0.2ᵃ	2.5 ± 0.5ᵇ
Metaxylem vessels (number)	5.8 ± 0.4ᵃ	5.1 ± 0.2ᵃ	3.5 ± 0.6ᵇ
Vessel diameter (µm)	28 ± 2ᵃ	25.1 ± 1.2ᵇ	18 ± 2.5ᶜ
Xylem area (µm^2^)	3,650 ± 280ᵃ	3,280 ± 320ᵇ	1,840 ± 300ᶜ
XAR (%)	86.9	79.4	69.4
WRI	0.08	0.09	0.21

Data are presented as mean ± standard error (x ± SE). Different letters within the same row indicate statistically significant differences between treatments, assessed by Tukey’s post hoc test (p < 0.05) following one-way analysis of variance (ANOVA). XAR values were calculated from the mean morphometric parameters of xylem and stele area

Thus, morphometric analysis demonstrated that increasing Pb concentrations were associated with reductions in the proportions of stele and xylem within the overall root structure, increased cortical porosity, enhanced endodermal differentiation, and increased the relative thickness of vessel walls. The most pronounced changes were observed at 0.5 mM Pb, indicating a profound reorganisation of the root epidermal, ground, and vascular tissues.

**Fig. 5:**
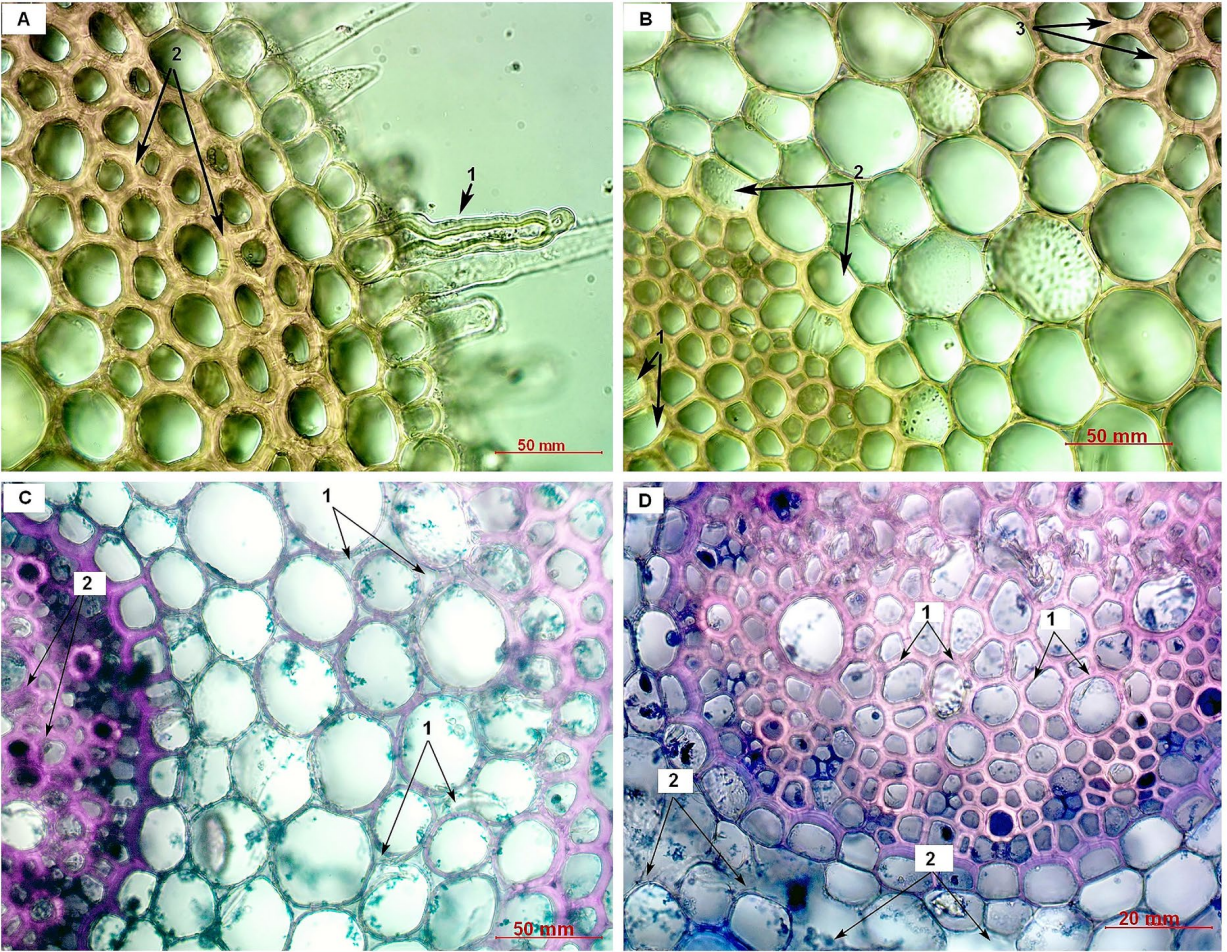
Anatomical structure of roots of 14-day-old *Triticum aestivum* seedlings under Pb exposure. (A) Control: 1 — root hairs; 2 — epidermis. (B) Control: 1 — xylem; 2 — endodermal cells; 3 — cortical parenchyma cells. (C) Roots exposed to 0.5 mM Pb^2+^ 1 — intercellular spaces; 2 — xylem cells. (D) Roots exposed to 0.5 mM Pb^2+^: 1 — xylem cells; 2 — intercellular spaces. Pb treatment induced pronounced structural alterations in the epidermal, cortical, and vascular tissues of wheat roots

**Fig. 6:**
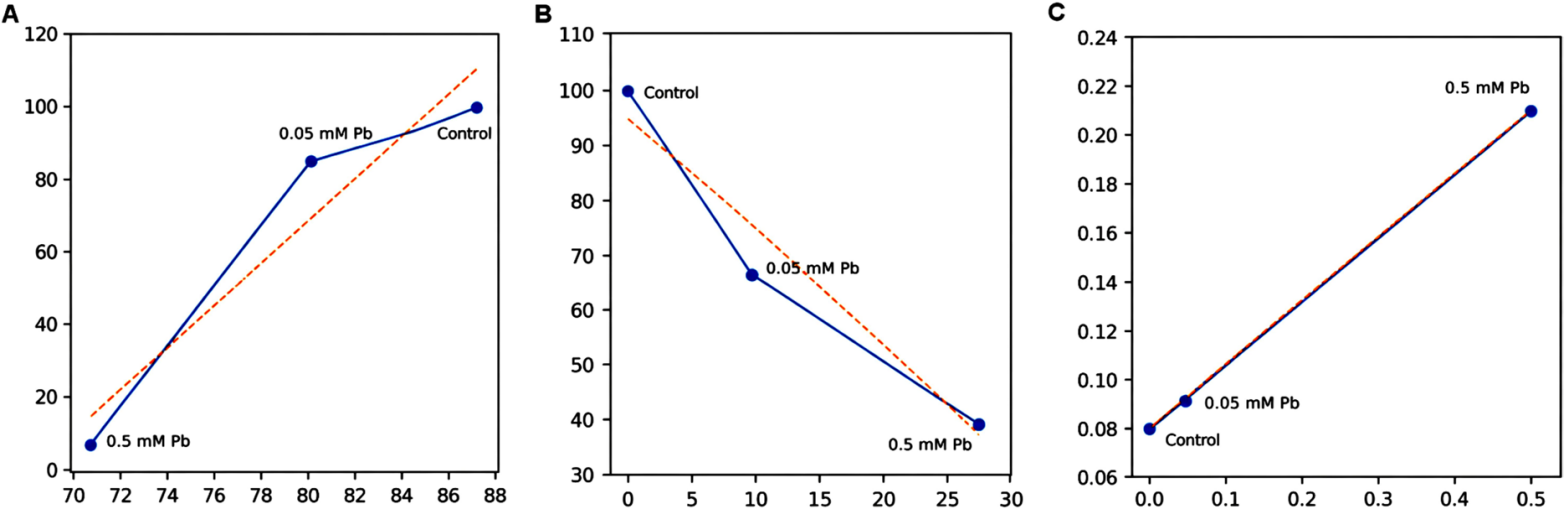
Relationships between anatomical traits and morphophysiological indices in 14-day-old *Triticum aestivum *L. seedlings exposed to Pb^2+^ treatments. Notes: (A) Relationship between xylem area ratio (XAR, %, x-axis) and root length (% of control, y-axis), R^2^ = 0.906; (B) relationship between cortex porosity index (CPI, %, x-axis) and root dry mass (% of control, y-axis), R^2^ = 0.910; (C) relationship between wall reinforcement index (WRI, y-axis) and Pb^2+^ concentration (mM, x-axis), R^2^ = 1.000. Points correspond to control plants and seedlings exposed to 0.05 and 0.5 mM Pb^2+^

The analysis of morphometric indices of root anatomical structure and morphophysiological parameters in wheat seedlings revealed consistent dose-dependent changes (Fig. 5, 6). In particular, the decrease in the proportion of xylem within the stele (XAR) from 86.9% in the control to 79.4% under 0.05 mM Pb^2+^ and 69.4% at 0.5 mM Pb was accompanied by reductions in root length of 13.1% and 93.8%, respectively. This pattern indicates a direct relationship between the reduction in conductive tissue and inhibition of root linear growth. In parallel, the increase in the cortical porosity index (CPI) from an undetectable level in the control to 8 ± 2% at 0.05 mM and 28 ± 5% at 0.5 mM Pb corresponded to decreases in root dry mass of 33.3% and 60.2%, respectively, suggesting a link between cortical parenchyma disorganisation and reduced biomass accumulation. At the same time, the vessel reinforcement index (WRI), which increased with toxicant concentration, was associated with a decrease in root fresh mass, reaching 28.4 ± 1.4 mg per plant at 0.5 mM Pb^2+^ (77.1% lower than the control). The increase in WRI reflects an enhanced relative thickness of vessel cell walls, accompanied by reduced vessel diameter, indicating the formation of a more rigid but less functionally efficient transport system.

Thus, the results demonstrate a clear correspondence between structural changes in the root (decreased XAR, increased CPI and WRI) and the suppression of morphophysiological parameters, suggesting that root anatomical reorganisation is a key mechanism underlying the toxic effect of lead in wheat plants.

## Discussion

The results should be interpreted in the context of agro-landscapes affected by missile strikes outside active combat zones. Such areas are often returned to agricultural use; however, they may retain hidden geochemical and physicochemical disturbances. Missile strikes affect soils through several complementary pathways. The detonation produces large quantities of fine particulate matter enriched with metals from the missile casing, engine components, warhead materials, and combustion products. These particles are deposited directly onto the soil surface and may subsequently migrate within the soil profile under the influence of precipitation and biological activity. Simultaneously, the blast wave mechanically disrupts the soil profile, mixes soil horizons, alters aggregate structure, and redistributes previously accumulated contaminants. Atmospheric deposition of explosion-derived aerosols further contributes to localised contamination. The relative importance of these pathways depends on the type of missile, explosive composition, impact energy, soil characteristics, and post-impact weather conditions. Consequently, missile strikes induce both direct chemical contamination and indirect physical degradation of soil, which together determine long-term soil health and ecosystem recovery.

Elevated concentrations of Pb, Zn, Cu, and Cr in soils from explosion craters, together with decreased pH and altered particle-size distribution, indicate the formation of local technogenic geochemical anomalies. Pollution indices further support this interpretation: the calculated Igeo for Pb falls within the “moderately to strongly contaminated” category, while EF values indicate significant anthropogenic enrichment. Similar effects have been reported in other rocket- and artillery-impacted areas in Ukraine, confirming that such disturbances are not isolated but may be widespread in post-conflict agricultural landscapes (Krainiuk et al., 2025; Petrushka et al., 2024). More broadly, military activities are increasingly recognised as an important source of trace-metal contamination in soils, particularly through explosive residues, combustion products, and the redistribution of contaminated fine particles (Broomandi et al., 2020; Leal Filho et al., 2024a,b; Solokha et al., 2024).

Measurements of gamma-ray emitter content revealed no statistically significant differences between samples taken from impact craters and control soil samples. The activity levels of naturally occurring radioactive substances in this region are comparable to those observed in other parts of the country (Menshikova et al., 2021; Popovych et al., 2022), and do not exceed global mean values (UNSCEAR, 2000). A thorough examination of the available data has revealed that the deployment of conventional munitions does not result in an elevated radiation risk. The underlying reason for this phenomenon is the exposure of deeper layers of the subsoil, which exhibit a distinct concentration of radioactive gamma-emitting radionuclides. The Gamma Level Index excesses were found to be insignificant within the range of 2–12%, thus indicating a relatively low to moderate health risk. With regard to the AGDE parameter, the global average (300 μSv year^−1^) was exceeded in 11 cases. However, the contribution of these doses to the total annual natural radiation dose for the Dnipropetrovsk region citizen (3.5 μSv) ranged from 5.71% to 14.3%. It can thus be concluded that the presence of NORM in the soil samples does not constitute a significant radiological risk. The analyses carried out have demonstrated that the soils under consideration, despite exhibiting locally elevated levels of radionuclides (most notably thorium-232 and potassium-40), do not constitute a radiological hazard to human health. In contrast to radionuclides, Pb was consistently enriched in the missile-affected soils and was among the principal contaminants identified in the crater zones. Therefore, Pb was selected as a representative model toxicant for controlled hydroponic experiments designed to investigate the biological consequences of metal exposure under defined conditions, while avoiding the confounding effects of heterogeneous field soils and mixed contaminants.

The observed decrease in soil pH is particularly significant because it may increase the mobility of metals and, consequently, their bioavailability to plants (Hou et al., 2020; Kabata-Pendias, 2011). Although lead in soils is often present in relatively immobile forms, changes in soil acidity and structure may promote partial transformation into more mobile fractions (Sintorini et al., 2021; Zhao et al., 2022). In addition, soil compaction and changes in particle-size distribution likely reduce aeration and water permeability, creating unfavourable conditions for root development. These processes align with elevated Igeo and EF values, which reflect not only increased concentrations but also the contaminants' anthropogenic origin and potential ecological relevance. The combined chemical and physical degradation of soil properties suggests that explosive-impact disturbances may persist beyond the immediate event, continuing to influence agroecosystem functioning. Such long-term persistence of technogenic soil alteration has been reported in other disturbed environments, including areas affected by fire, industrial deposition, and military activity (Certini et al., 2013; Solokha et al., 2024).

It is important to emphasise that total Pb in soil does not directly reflect plant uptake, as bioavailability is governed by pH, organic matter, carbonates, and competing ions (Kabata-Pendias, 2011). Nevertheless, the recorded Pb concentrations in crater soils indicate a substantial contaminant pool relative to nearby control soils. In this context, *T. aestivum* serves as a sensitive indicator of soil contamination, enabling extrapolation of laboratory findings to field conditions. At the same time, the hydroponic Pb treatments should be understood as a controlled exposure gradient designed to model biologically relevant stress responses rather than to directly reproduce total soil concentrations. This approach is consistent with experimental studies showing that plant responses to metals depend strongly on toxicant speciation and mobility rather than on bulk concentration alone (Kabata-Pendias, 2011; Zhao et al., 2022; Gupta et al., 2024).

At the tissue level, lead induced pronounced reorganisation of root structure. At 0.05 mM, enhanced suberisation and lignification of the endodermis were observed, which can be interpreted as an adaptive barrier mechanism aimed at limiting apoplastic metal transport (Enstone et al., 2002; Schreiber et al., 1999). However, at 0.5 mM, these mechanisms were insufficient: tissue disorganisation, deformed cells, and disruption of endodermal integrity were observed, indicating severe stress-induced damage (Poschenrieder et al., 2008). The increase in CPI indicates degradation of the cortical parenchyma, while the reduction in biomass confirms functional impairment of the tissues. A decrease in XAR, a reduction in vessel diameter, and an increase in WRI characterised changes in the xylem. This pattern reflects the formation of a more mechanically reinforced but less efficient transport system, consistent with the trade-off between hydraulic safety and efficiency (Hacke et al., 2001; Tyree & Zimmermann, 2002;). Similar xylem and endodermal responses have been described in plants exposed to heavy metal stress, where structural reinforcement is often accompanied by reduced transport capacity and growth suppression (Schreiber et al., 1999; Enstone et al., 2002). The reduction in functional xylem area was directly associated with inhibited root growth, indicating that limited hydraulic conductivity is a key mechanism of lead toxicity.

A strong correspondence was observed between anatomical indices (XAR, CPI, WRI) and morphophysiological parameters. A proportional reduction in root length and biomass accumulation accompanied decreases in XAR and increases in CPI and WRI. This suggests that root structural reorganisation is a key mechanism underlying the toxic action of lead, resulting in functional limitations in growth and transport processes. Similar relationships between root anatomy and performance under metal stress have been documented in wheat and other crop species, where anatomical remodelling may initially act as a defence response but becomes maladaptive at higher exposure levels (Hacke et al., 2001; Tyree & Zimmermann, 2002). In this respect, anatomical traits appear to be sensitive indicators of stress intensity and may be useful for detecting early dysfunction in contaminated agroecosystems.

Our elevated root Pb concentrations, with limited shoot translocation, align with established plant defence mechanisms. Pb stress triggers cell wall remodelling, increasing low-methylesterifiedpectins that sequester metal ions (Krzesłowka et al., 2013). These pectin modifications, observed across roots, protonemata, and fronds, complement general heavy-metal tolerance strategies (Fahr et al., 2013; Kosakivska et al., 2021; Krzesłowka et al., 2013). This explains the restricted Pb mobility in Ukrainian war-affected plants despite the extreme soil contamination levels reported in the region (Krainiuk et al. 2025; Solokha et al. 2024).

Taken together, the results indicate that the effects of missile-derived contamination are realised through integrated changes at the soil level, in root tissues, and in the vascular system. The observed pattern suggests that even partially remediated or visually recovered areas may remain biologically hazardous, as soil-bound Pb may be mobilised under changing physicochemical conditions. This highlights the importance of long-term monitoring and integrated assessment of technogenically disturbed agro-landscapes before their safe reintroduction to agricultural use. These findings also underscore the need to establish long-term monitoring stations to track temporal changes in soil physicochemical properties, contaminant dynamics, and soil recovery after missile strikes. Such monitoring would improve understanding of the persistence of military-derived contamination and support evidence-based land management and remediation in post-conflict agricultural areas. Overall, root structural reorganisation appears to be a central component of the response to Pb stress and helps explain the reduction in hydraulic efficiency and biomass accumulation observed in wheat seedlings. These findings also suggest that root anatomical traits may serve as potential early indicators of contamination-related stress in crop plants, particularly in post-conflict agricultural systems where conventional visual assessment may underestimate ecological risk (Krainiuk et al., 2025; Solokha et al., 2024).

## Conclusion

This study demonstrates the impact of the missile strikes on agricultural landscapes outside active combat zones. It was found that the missile strikes can cause significant degradation of soil physicochemical properties and acute metals contamination. Explosion craters exhibited elevated concentrations of Pb, Zn, Cu, Mn, and Cr, exceeding both natural background levels and regulatory thresholds. These findings confirm the formation of localized technogenic geochemical anomalies driven by ammunition detonation and infrastructure destruction. Furthermore, observed soil acidification, increased compaction, and altered particle-size distribution likely enhance the mobility and bioavailability of these toxic elements.

Using gamma – ray spectrometer (equipped with the HPGe detector), the specific activity of NORM radionuclides (Ra-226, Th-232, K-40) and caesium-137 was determined. The activity of the Cs-137 isotope was found to be highest (53.70 Bq kg^−1^) in sample 4’, and lowest (0.34 Bq kg^−1^) in sample 2. The range of natural ^40^ K levels was from 276.9 Bq kg^−1^ (sample 4’) to 640.6 Bq/kg (sample 7). A noteworthy correlation was identified, in conditions of low potassium concentration, a higher radiocaesium content was recorded. This phenomenon may be attributed to the biological capacity of plants to accumulate Cs under conditions of potassium deficiency. ^232^Th exhibited higher activity than Ra-226 in almost all samples. This phenomenon may be due to the fact that radium is a calcium analogue and is sometimes absorbed by plants, leading to its redistribution. The radium activity equivalent (Ra_eq_) ranged from 58.68 to 150.2 Bq kg^−1^ and was well below the safety threshold (370 Bq kg^−1^) in all cases. The gamma index exceeded the limit value (1.0) in eight samples (the excesses were found to be within the range of 2–12%). In the AGDE parameter case, the values calculated for the soil samples ranged from 199.7 to 500.5 μSv year^−1^, indicating low to medium health risk. The ELCR analysis lead to the conclusion that the global average (0.29∙10⁻^3^) was exceeded in nine samples. The highest value (0.35∙10⁻^3^ for samples 3 and 7), was only marginally higher than this figure.

Phytotoxicity experiments using *T, aestivum* revealed a clear dose-dependent response to lead. While a slight hormetic effect was noted at low concentrations, higher levels resulted in severe growth retardation, reduced biomass accumulation, and strong negative correlations between Pb concentration and morphophysiological traits.

Anatomical analysis of the root system identified profound structural reorganization as a primary mechanism of Pb toxicity. Key modifications included:a reduced proportion of stele and xylem, increased cortical porosity, enhanced suberization and lignification of the endodermis, and diminished vascular efficiency.

These structural disruptions directly correlate with impaired growth performance. Ultimately, these results underscore the persistent ecological legacy of military activity. The findings emphasize the critical necessity for systematic monitoring and remediation of contaminated agroecosystems to ensure their safe reintegration into food production.

## Data Availability

The data that support the findings of this study are available from the corresponding author.

## References

[CR1] Abràmoff, M. D., Magalhães, P. J., & Ram, S. J. (2004). Image processing with ImageJ. *Biophotonics International,**11*(7), 36–42. 10.1111/j.1365-2818.2004.01265.x

[CR2] Amin, H., Arain, B. A., Amin, F., & Surhio, M. A. (2013). Phytotoxicity of chromium on germination, growth and biochemical attributes of *Hibiscus esculentus* L. *American Journal of Plant Sciences,**4*, 2431–2439. 10.4236/ajps.2013.412302

[CR3] Baliuk, S. A., Kucher, A. V., Solokha, M. O., & Solovei, V. B. (2024). Assessment of the impact of armed aggression of the RF on the soil cover of Ukraine. *Ukrainian Geographical Journal,**1*, 7–18. 10.15407/ugz2024.01.007

[CR4] Baluska, F., Volkmann, D., & Barlow, P. W. (2013). Preparation of root sections for light microscopy. In P. W. Barlow (Ed.), *Root development: Methods and protocols* (Vol. Vol. 959, pp. 117–128). Humana Press. 10.1007/978-1-62703-207-2_9

[CR5] Battey, N. H. (2003). October - The unnatural knowledge of colour. *Journal of Experimental Botany,**54*, 2197–2200. 10.1093/jxb/erg260 14504295 10.1093/jxb/erg260

[CR6] Bilyi, T., Hlavatskyi, D., Poliachenko, I., Melny, G., Cherkes, S., & Litvinov, D. (2025). The degree of soil degradation and aerosol formation from explosion products resulting from hostilities in Ukraine. *Visnyk of Taras Shevchenko National University of Kyiv: Geology,**1*(108), 39–46. 10.7721/1728-2713.108.05

[CR7] Broomandi, P., Guney, M., Kim, J. R., & Karaca, F. (2020). Soil contamination in areas impacted by military activities: A critical review. *Sustainability,**12*, Article 9002. 10.3390/su12219002

[CR8] Brundrett, M. C., Enstone, D. E., & Peterson, C. A. (1988). A berberine-aniline blue fluorescent staining procedure for suberin, lignin, and callose in plant tissue. *Protoplasma,**146*(2), 133–142.

[CR9] Bulubasa, B., Costinel, D., Miu, A. F., & Ene, M. R. (2021). Activity concentrations of ^238^U, ^232^Th, ^226^Ra, ^137^Cs and ^40^K radionuclides in honey samples from Romania: Lifetime cancer risk estimated. *Journal of Environmental Radioactivity,**234*, Article 106626. 10.1016/j.jenvrad.2021.106626 33940545 10.1016/j.jenvrad.2021.106626

[CR10] Certini, G., Scalenghe, R., & Woods, W. I. (2013). The impact of warfare on the soil environment. *Earth-Science Reviews,**127*, 1–15. 10.1016/j.earscirev.2013.08.009

[CR11] Darby, S., Hill, D., Auvinen, A., Barros-Dios, J.M., Baysson, H., Bochicchio, F., Deo, H., Falk, R., Forastiere, F., Hakama, M., Heid, I., Kreienbrock, L., Kreuzer, M., Lagarde, F., Mäkeläinen, I., Muirhead, C., Oberaigner, W., Pershagen, G., Ruano-Ravina, A., Ruosteenoja, E., Schaffrath Rosario, A., Tirmarche, M., Tomásek, L., Whitley, E., Wichmann, H-E., Doll, R. (2005). Radon in homes and risk of lung cancer: collaborative analysis of individual data from 13 European case-control studies. *BMJ*, 330(7485):223. 10.1136/bmj.38308.477650.63 10.1136/bmj.38308.477650.63 PMC546066 15613366

[CR12] Enstone, D. E., Peterson, C. A., & Ma, F. (2002). Root endodermis and exodermis: Structure, function, and responses to the environment. *Journal of Plant Growth Regulation,**21*, 335–351. 10.1007/s00344-003-0002-2

[CR13] European Commission, Joint Research Centre, European Soil Data Centre (ESDAC). (2012). Soil contamination and soil protection. https://esdac.jrc.ec.europa.eu/themes/soil-contamination (accessed 30 June 2026)

[CR14] Fahr, M., Laplaze, L., Bendaou, N., et al. (2013). Effect of lead on root growth. *Frontiers in Plant Science,**4*, 175. 10.3389/fpls.2013.00175 23750165 10.3389/fpls.2013.00175 PMC3674728

[CR15] Fijałkowski, K., Kacprzak, M., Grobelak, A., & Placek, A. (2012). The influence of selected soil parameters on the mobility of heavy metals in soils. *Inżynieria i Ochrona Środowiska,**15*, 81–92.

[CR16] Food and Agriculture Organization of the United Nations (FAO). (2022). FAO's engagement in Ukraine. https://www.fao.org/family-farming/detail/en/c/1476928/ (accessed 30 June 2026)

[CR17] Gupta, M., Dwivedi, V., Kumar, S., Patel, A., Niazi, P., & Yadav, V. K. (2024). Lead toxicity in plants: Mechanistic insights into toxicity, physiological responses of plants and mitigation strategies. *Plant Signaling & Behavior,**19*(1), Article 2365576. 10.1080/15592324.2024.2365576 38899525 10.1080/15592324.2024.2365576 PMC11195469

[CR18] Hacke, U. G., Sperry, J. S., Pockman, W. T., Davis, S. D., & McCulloh, K. A. (2001). Trends in wood density and structure are linked to prevention of xylem implosion by negative pressure. *Oecologia,**126*, 457–461. 10.1007/s004420100628 28547229 10.1007/s004420100628

[CR19] Hlavatskyi, D., Bonchkovskyi, O. S., Ostapenko, P., Bondar, K. M., Bakhmutov, V. G., Bonchkovskyi, A., Menshov, O., Poliachenko, I., & Shvaiko, V. (2025). Classification of war-induced soil contamination by the type of military impact in Eastern Ukraine. *Land Degradation & Development*. 10.1002/ldr.70397

[CR20] Hou, D., O’Connor, D., Igalavithana, A. D., Igalavithana, A. D., Alessi, D. S., Luo, J., Tsang, D. C. W., Sparks, D. L., Yamauchi, Y., Rinklebe, J., & Ok, Y. S. (2020). Metal contamination and bioremediation of agricultural soils for food safety and sustainability. *Nature Reviews Earth & Environment,**1*, 366–381. 10.1038/s43017-020-0061-y

[CR21] Hryhorczuk, D., Levy, B. S., Prodanchuk, M., et al. (2024). The environmental health impacts of Russia’s war on Ukraine. *Journal of Occupational Medicine and Toxicology,**19*, Article 1. 10.1186/s12995-023-00398-y 38183124 10.1186/s12995-023-00398-y PMC10768292

[CR23] Illienko, V., Salnikova, A., Klepko, A., & Lazarev, M. (2025). Military soil degradation in the northern part of Ukraine. *EGU General Assembly 2025*, EGU25-8455. 10.5194/egusphere-egu25-8455

[CR24] Kabata-Pendias, A. (2011). *Trace elements in soils and plants* (4th ed.). CRC Press.

[CR25] Kaissas, I., Clouvas, A., Postatzii, M., Xanthos, S., & Omirou, M. (2023). Long-term study (1987–2023) on the distribution of ^137^Cs in soil following the Chernobyl nuclear accident: A comparison of temporal migration measurements and compartment model predictions. *Radiation Protection Dosimetry,**199*(19), 2366–2372. 10.1093/rpd/ncad241 37698137 10.1093/rpd/ncad241 PMC10655059

[CR26] Kaur, G., Singh, H. P., Batish, D. R., & Kohli, R. K. (2013). Lead (Pb)-induced biochemical and ultrastructural changes in wheat (*Triticum aestivum*) roots. *Protoplasma,**250*, 53–62. 10.1007/s00709-011-0372-4 22231903 10.1007/s00709-011-0372-4

[CR27] Kosakivska, I. V., Babenko, L. M., Romanenko, K. O., Korotka, I. Y., & Potters, G. (2021). Molecular mechanisms of plant adaptive responses to heavy metal stress. *Cell Biology International,**45*, 258–272. 10.1002/cbin.11503 33200493 10.1002/cbin.11503

[CR28] Kosakivska, I. V., Babenko, L. M., Voytenko, L. V., Vasyuk, V. A., Shcherbatiuk, M. M., & Romanenko, K. O. (2025). Natural growth regulators in enhancing cereal crop resistance to lead contamination. *Cereal Research Communications,**53*, 2063–2074. 10.1007/s42976-025-00697-6

[CR29] Kouroukla, E., Gooding, T. D., & Fonseca, H. S. (2024). Analysis of radon mitigation methods: 10-year review. *Journal of Radiological Protection,**44*, Article 031503. 10.1088/1361-6498/ad58e8 10.1088/1361-6498/ad58e8 38885627

[CR30] Krainiuk, O. V., Buts, Y. V., Ponomarenko, R. V., Asotskyi, V. V., & Darmofal, E. A. (2025). Environmental consequences of military operations in Ukraine on the example of soil research in the Kharkiv region. *Journal of Geology, Geography and Geoecology,**34*, 304–317. 10.15421/112526

[CR31] Krzesłowska, M., Rabęda, I., Lewandowski, M., et al. (2013). Pb induces plant cell wall modifications—in particular—the increase of pectins able to bind metal ions level. *E3S Web of Conferences, 1*, 26008. 10.1051/e3sconf/20130126008.

[CR32] Kumar, S., & Misra, A. N. (2023). Effect of lead stress on morphological and physiological features of wheat (*Triticum aestivum* L.) during vegetative stage. *Biological Forum – An International Journal, 15*(10), 918–924.

[CR33] Lamhamdi, M., El Galiou, O., Bakrim, A., Novoa-Munoz, J. C., Arias-Estevez, M., Aarab, A., & Lafont, R. (2013). Effect of lead stress on mineral content and growth of wheat (*Triticum aestivum*) and spinach (*Spinacia oleracea*) seedlings. *Saudi Journal of Biological Sciences,**20*(1), 29–36. 10.1016/j.sjbs.2012.09.001 23961216 10.1016/j.sjbs.2012.09.001 PMC3730938

[CR34] Leal Filho, W., Eustachio, J. H. P. P., Fedoruk, M., & Lisovska, T. (2024a). War in Ukraine: An overview of environmental impacts and consequences for human health. *Frontiers in Sustainable Resource Management,**3*, Article 1423444. 10.3389/fsrma.2024.1423444

[CR35] Leal Filho, W., Fedoruk, M., Eustachio, J. H. P. P., Splodytel, A., Smaliychuk, A., & Szynkowska-Jóźwik, M. I. (2024b). The environment as the first victim: The impacts of the war on the preservation areas in Ukraine. *Journal of Environmental Management,**364*, Article 121399. 10.1016/j.jenvman.2024.121399 38878570 10.1016/j.jenvman.2024.121399

[CR36] Liu, Y., Wen, L., Yu, Q., Li, J., Hou, H., Cao, Y., & Wu, X. (2026). Evolution and future path of global farmland soil pollution remediation over the past five decades. *Integrated Environmental Assessment and Management*. 10.1093/inteam/vjag008 10.1093/inteam/vjag008 41830145

[CR37] Menshikova, E., Perevoshchikov, R., Belkin, P., & Blinov, S. (2021). Concentrations of natural radionuclides (^40^K, ^226^Ra, ^232^Th) at the potash salts deposit. *Journal of Ecological Engineering,**22*(3), 179–187. 10.12911/22998993/132544

[CR38] Ministry of Economy of Ukraine. (n.d.). State register of plant varieties suitable for dissemination in Ukraine. https://me.gov.ua/Documents/Detail?lang=uk-UA&id=c5e26c83-ac95-43b8-8d53-a5f8f907099f&title=DerzhavniiRestrSortiv-PridatnikhDliaPos (accessed 30 June 2026)

[CR39] Müller, G. (1969). Index of geoaccumulation in sediments of the Rhine River. *GeoJournal,**2*, 108–118.

[CR40] Müller, G. (1981). The heavy metal pollution of the sediments of Neckar and its tributary: A stocktaking. *Chemiker-Zeitung,**105*, 157–164.

[CR41] Munitions and chemicals: How does war damage soils, and what are the solutions? (2022, October 13). https://rubryka.com/en/article/soil-ukraine/ (accessed 30 June 2026)

[CR42] Mystrioti, C., & Papassiopi, N. (2024). A comprehensive review of remediation strategies for soil and groundwater contaminated with explosives. *Sustainability,**16*(3), 961. 10.3390/su16030961

[CR43] Paksoy, M., & Acar, B. (2009). Effect of organic fertilizers on yield components of some tomato cultivars. *Asian Journal of Chemistry,**21*(8), 6041–6047.

[CR44] Petrushka, K., Malovanyy, M., Skrzypczak, D., Chojnacka, K., & Warchoł, J. (2024). Risks of soil pollution with toxic elements during military actions in Lviv. *Journal of Ecological Engineering*. 10.12911/22998993/175136

[CR45] Piermattei, A., von Arx, G., Avanzi, C., Fonti, P., Gärtner, H., Piotti, A., Urbinati, C., Vendramin, G. G., Büntgen, U., & Crivellaro, A. (2020). Functional relationships of wood anatomical traits in Norway spruce. *Frontiers in Plant Science,**11*, Article 683. 10.3389/fpls.2020.00683 32528514 10.3389/fpls.2020.00683 PMC7266088

[CR46] Popovych, V., Henyk, Y., Gapalo, A., Bosak, P., & Popovych, N. (2022). Specific activity of radionuclides in soils disturbed by forest fires. *Journal of Ecological Engineering,**23*(6), 265–270. 10.12911/22998993/148191

[CR47] Poschenrieder, C., Gunsé, B., Corrales, I., & Barceló, J. (2008). A glance into aluminum toxicity and resistance in plants. *Journal of Plant Nutrition and Soil Science,**171*(2), 185–197. 10.1002/jpln.200700049 10.1016/j.scitotenv.2008.06.003 18657304

[CR48] Priyadarshanee, M., Mahto, U., & Das, S. (2022). Mechanism of toxicity and adverse health effects of environmental pollutants. *Microbial biodegradation and bioremediation* (pp. 33–53). Elsevier. 10.1016/b978-0-323-85455-9.00024-2

[CR49] Rani, M., Vikas, Kumar, R., Lathwal, M., Kamboj, A. (2024). Effect and Responses of Lead Toxicity in Plants. In: Kumar, N., Jha, A.K. (eds) Lead Toxicity Mitigation: Sustainable Nexus Approaches. Environmental Contamination Remediation and Management. Springer, Cham. 10.1007/978-3-031-46146-0.

[CR50] Rani, M., Vikas, Kumar, R., Lathwal, M., & Kamboj, A. (2024). Effect and responses of lead toxicity in plants. In N. Kumar & A. K. Jha (Eds.), *Lead toxicity mitigation: Sustainable nexus approaches* (pp. 211–241). Springer. 10.1007/978-3-031-46146-0_10

[CR51] Ratnayaka, H. H., Molin, W. T., & Sterling, T. M. (2003). Physiological and antioxidant responses of cotton and spurred anoda under interference and mild drought. *Journal of Experimental Botany,**54*, 2293–2305. 10.1093/jxb/erg251 14504299 10.1093/jxb/erg251

[CR52] Schreiber, L., Hartmann, K., Skrabs, M., & Zeier, J. (1999). Apoplastic barriers in roots: Chemical composition of endodermal and hypodermal cell walls. *Journal of Experimental Botany,**50*, 1267–1280. 10.1093/jxb/50.337.1267

[CR53] Sintorini, M., Widyatmoko, H., Sinaga, E., & Aliyah, N. (2021). Effect of pH on metal mobility in the soil. *IOP Conference Series: Earth and Environmental Science,**737*, Article 012071. 10.1088/1755-1315/737/1/012071

[CR54] Skalny, A. V., Aschner, M., Bobrovnitsky, I. P., et al. (2021). Environmental and health hazards of military metal pollution. *Environmental Research,**201*, Article 111568. 10.1016/j.envres.2021.111568 34174260 10.1016/j.envres.2021.111568

[CR55] Solokha, M., Demyanyuk, O., Symochko, L., Mazur, S., Vynokurova, N., Sementsova, K., & Mariychuk, R. (2024). Soil degradation and contamination due to armed conflict in Ukraine. *Land,**13*, Article 1614. 10.3390/land13101614

[CR56] Szaciłowski, G. (2024). Evaluation of natural radioactivity and assessment of dose increase due to the use of fertilizers produced in Poland. *Journal of Radioanalytical and Nuclear Chemistry,**333*, 3425–3430. 10.1007/s10967-023-09241-4

[CR57] Thomas, G. W. (1996). Soil pH and soil acidity. In D. L. Sparks et al. (Eds.), *Methods of soil analysis: Part 3 chemical methods* (SSSA Book Series 5.3). Soil Science Society of America. 10.2136/sssabookser5.3.c16

[CR58] Tóth, G., Hermann, T., Da Silva, M. R., & Montanarella, L. (2016). Heavy metals in agricultural soils of the European Union with implications for food safety. *Environment International,**88*, 299–309. 10.1016/j.envint.2015.12.017 26851498 10.1016/j.envint.2015.12.017

[CR59] Tyree, M. T., & Zimmermann, M. H. (2002). Xylem structure and the ascent of sap. *Springer*. 10.1007/978-3-662-04931-0

[CR60] United States Environmental Protection Agency. (2023). Regional screening levels (RSLs) for chemical contaminants at Superfund sites. https://www.epa.gov/risk/regional-screening-levels-rsls (accessed 30 June 2026)

[CR61] UNSCEAR. (2000). *Source and effects of ionising radiation: Report to the General Assembly with scientific annexes*. United Nations.

[CR62] Wan, Y., Liu, J., Zhuang, Z., Wang, Q., & Li, H. (2024). Heavy metals in agricultural soils: Sources, influencing factors, and remediation strategies. *Toxics,**12*(1), 63. 10.3390/toxics12010063 38251018 10.3390/toxics12010063 PMC10819638

[CR63] Welsh, C. (2024). Russia, Ukraine, and global food security: A two-year assessment. Center for Strategic and International Studies. https://www.csis.org/analysis/russia-ukraine-and-global-food-security-two-year-assessment

[CR64] Wilkins, D. A. (1957). A technique for measurement of lead tolerance in plants. *Nature,**180*(4575), 37–38. 10.1038/180037b013451634

[CR65] World Health Organization. (2009). *WHO handbook on indoor radon: a public health perspective*. ISBN: 9789241547673 23762967

[CR66] Yakymchuk, A., Balanda, O., & Bzowska-Bakalarz, M. (2024). Assessment of soil contamination of Ukraine with heavy metals during the war. *Scientific Papers of Silesian University of Technology - Organization and Management Series, 196*, 45. 10.29119/1641-3466.2024.196.45

[CR67] Yao, C., Yang, Y., Li, C., Shen, Z., Li, J., Mei, N., Luo, C., Wang, Y., Zhang, C., & Wang, D. (2024). Heavy metal pollution in agricultural soils from surrounding industries with low emissions: Assessing contamination levels and sources. *Science of the Total Environment,**917*, Article 170610. 10.1016/j.scitotenv.2024.170610 38307271 10.1016/j.scitotenv.2024.170610

[CR68] Yu, H. A., Nic Daeid, N., Dawson, L. A., DeTata, D. A., & Lewis, S. W. (2017). Explosive detonation causes an increase in soil porosity leading to increased TNT transformation. *PLoS ONE,**12*(12), Article e0189177. 10.1371/journal.pone.0189177 29281650 10.1371/journal.pone.0189177 PMC5744939

[CR69] Zakaly, H. M. H., Abbasi, A., Almousa, N., & Savasan, A. (2024). Naturally occurring radioactive materials (NORM) concentration and health risk assessment of aerosols dust in Nicosia, North Cyprus. *Journal of Radioanalytical and Nuclear Chemistry,**333*, 1073–1082. 10.1007/s10967-023-09346-w

[CR70] Zhao, H., Wu, Y., Lan, X., et al. (2022). Comprehensive assessment of harmful heavy metals in contaminated soil in order to score pollution level. *Scientific Reports,**12*, Article 3552. 10.1038/s41598-022-07602-9 35241759 10.1038/s41598-022-07602-9 PMC8894455

